# Myocardial infarction reduces cardiac nociceptive neurotransmission through the vagal ganglia

**DOI:** 10.1172/jci.insight.155747

**Published:** 2022-02-22

**Authors:** Siamak Salavatian, Jonathan D. Hoang, Naoko Yamaguchi, Zulfiqar Ali Lokhandwala, Mohammed Amer Swid, John Andrew Armour, Jeffrey L. Ardell, Marmar Vaseghi

**Affiliations:** 1 UCLA Cardiac Arrhythmia Center and; 2 UCLA Neurocardiology Research Program of Excellence, Los Angeles, California, USA.

**Keywords:** Cardiology, Neuroscience, Cardiovascular disease

## Abstract

Myocardial infarction causes pathological changes in the autonomic nervous system, which exacerbate heart failure and predispose to fatal ventricular arrhythmias and sudden death. These changes are characterized by sympathetic activation and parasympathetic dysfunction (reduced vagal tone). Reasons for the central vagal withdrawal and, specifically, whether myocardial infarction causes changes in cardiac vagal afferent neurotransmission that then affect efferent tone, remain unknown. The objective of this study was to evaluate whether myocardial infarction causes changes in vagal neuronal afferent signaling. Using in vivo neural recordings from the inferior vagal (nodose) ganglia and immunohistochemical analyses, structural and functional alterations in vagal sensory neurons were characterized in a chronic porcine infarct model and compared with normal animals. Myocardial infarction caused an increase in the number of nociceptive neurons but a paradoxical decrease in functional nociceptive signaling. No changes in mechanosensitive neurons were observed. Notably, nociceptive neurons demonstrated an increase in GABAergic expression. Given that nociceptive signaling through the vagal ganglia increases efferent vagal tone, the results of this study suggest that a decrease in functional nociception, possibly due to an increase in expression of inhibitory neurotransmitters, may contribute to vagal withdrawal after myocardial infarction.

## Graphical abstract

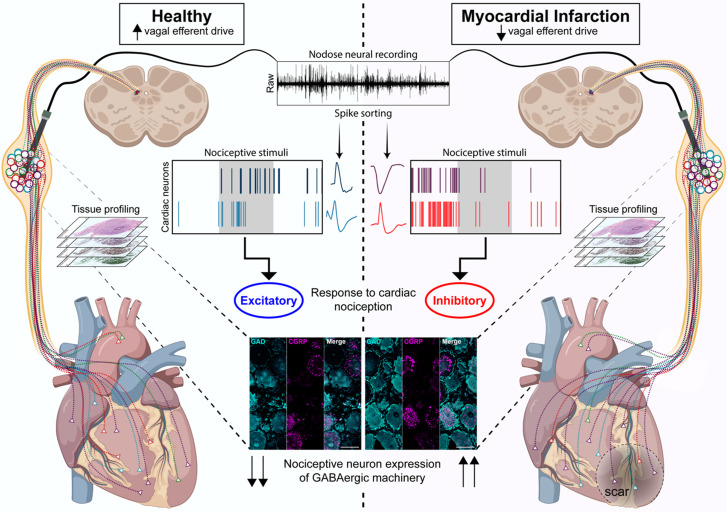

## Introduction

The autonomic nervous system plays an important role in the regulation of cardiac function (1, 2). Cardiac disease causes significant pathological changes in the autonomic nervous system that result in sympathovagal imbalance, as reflected by sympathetic activation and vagal withdrawal (3, 4). Notably, enhanced vagal drive has been shown to be antiarrhythmic, increasing cardiac action potential duration, slowing heart rate, preventing intracellular calcium overload, and reducing ventricular tachycardia (VT) inducibility (5, 6).

In the setting of myocardial injury, progressive vagal efferent dysfunction occurs, as reflected in noninvasive markers such as decreased heart rate variability. These changes are associated with an increased risk of ventricular arrhythmias in patients with myocardial infarction (MI) and increased mortality in heart failure (7, 8). The mechanisms underlying parasympathetic dysfunction and vagal withdrawal remain unclear.

In normal hearts, activation of cardiac afferent neurons, notably, vagal nociceptive neurons, leads to increased vagal efferent outflow (9). These pseudounipolar, cardiac specific sensory neurons reside in the inferior vagal (nodose) ganglia and transmit signals from the heart to the brainstem (10), subsequently modulating efferent vagal outflow to the heart (11). Increased vagal afferent nociceptive neurotransmission is associated with increased efferent vagal drive in normal hearts. However, no studies have evaluated changes in afferent vagal neurotransmission after cardiac disease to delineate abnormalities that may contribute to vagal dysfunction, and it is unknown if MI can cause extrathoracic vagal neuronal remodeling. In this study, we hypothesized that chronic MI causes changes in parasympathetic afferent neurotransmission. To understand these alterations, we probed structural and functional alterations in the nodose ganglia neurons in both normal and chronically infarcted porcine animals.

## Results

### Effect of MI on nodose neurons.

**Figure 1:**
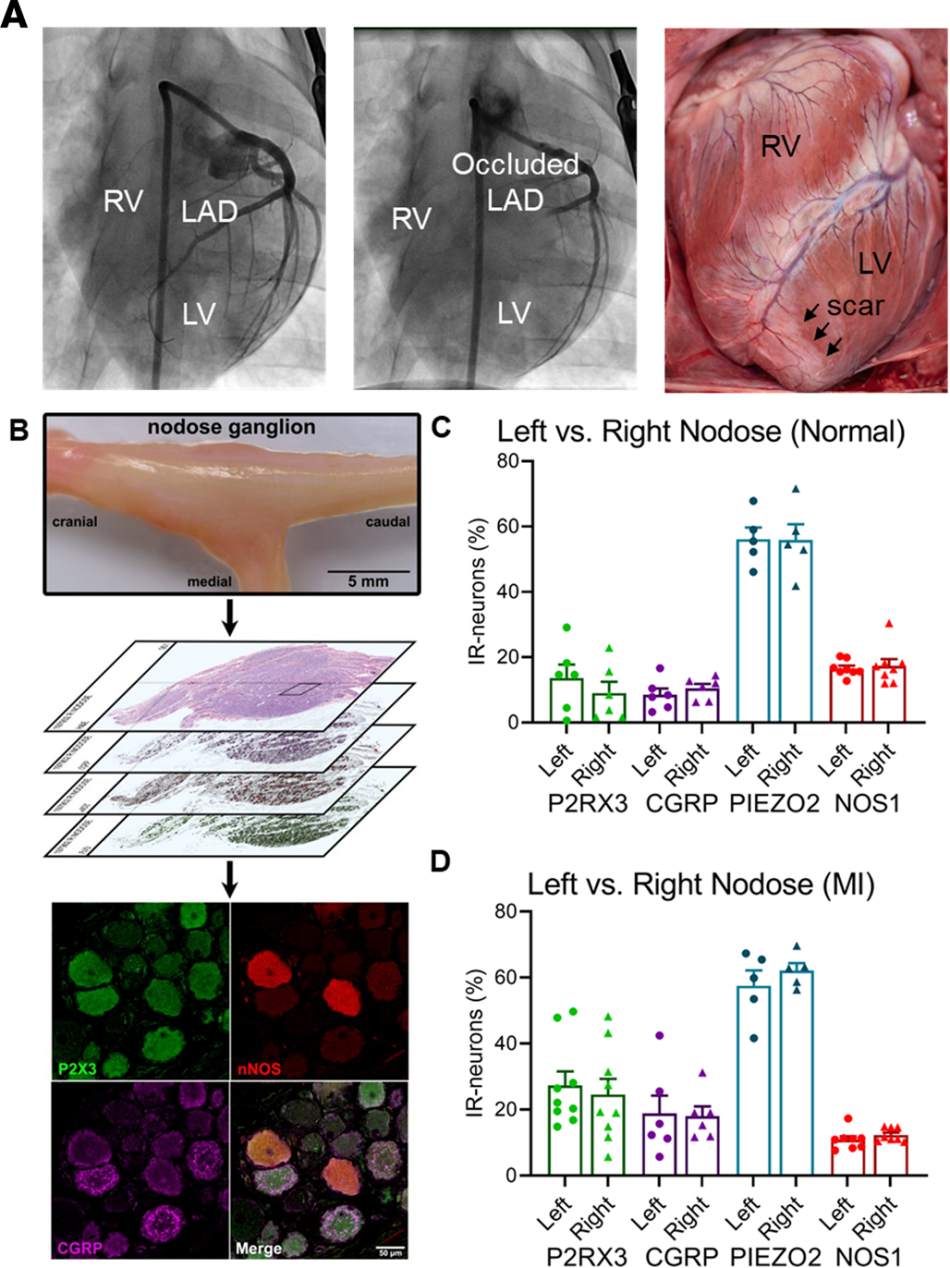
Assessment of expression profiles of left versus right nodose ganglia neurons in normal and LAD-infarcted animals. (**A**) Creation of myocardial infarct in the LAD distribution. Left panel: representative coronary angiography. Middle panel: Coronary artery angiography after microsphere injection showing occlusion at the mid-LAD. Right panel: Representative gross image of an infarcted heart showing scar in the region vascularized by the LAD. (**B**) Nodose ganglia from normal and infarcted animals were removed and analyzed for immunohistochemical changes. Scale bars are 5 mm (top), 50 μm (bottom). (**C**) Percentages of nodose ganglia neurons from normal and (**D**) infarcted (MI) animals expressing P2RX3, CGRP, PIEZO2, and NOS1 were quantified. No difference in the expression profiles of neurons between the left and right nodose ganglia was found in either normal or infarcted animals for P2RX3 (normal left vs. right nodose: *P* = 0.82; MI left vs. right nodose: *P* = 0.67), CGRP (normal left vs. right nodose: *P* = 0.82; MI left vs. right nodose: *P* = 0.89), PIEZO2 (a mechanosensitive ion channel; normal left vs. right nodose: *P* = 0.97; MI left vs. right nodose: *P* = 0.67), or NOS1 (normal left vs. right nodose: *P* = 0.82; MI left vs. right nodose: *P* = 0.67). *n* = 6 pairs of nodose for P2RX3 in normal animals and CGRP in both normal and MI animals. *n* = 5 pairs of nodose ganglia for PIEZO2 in normal and MI animals. *n* = 8 pairs of nodose ganglia for NOS1 in normal and MI animals. Data shown as mean  ±  SEM; paired, 2-tailed Student’s *t* test with the false discovery rate corrected by the Benjamini-Hochberg method was used for analyses. CGRP, calcitonin gene-related peptide; LV, left ventricle; NOS1, nitric oxide synthase 1; P2RX3, P2X purinoceptor 3; RV, right ventricle.

It is unknown if MI is associated with phenotypical changes in the neurons of the nodose ganglia. To assess these alterations after MI, ganglia were harvested from chronically infarcted animals as previously described (5, 12), then compared with ganglia from healthy animals. Morphological changes and protein expression profiles were assessed in animals with chronic MI with left anterior descending coronary artery (LAD) occlusion (Figure 1) and in animals with right coronary artery (RCA) infarction, to determine whether the location of the infarct affected the laterality of changes observed (i.e., in right vs. left vagal/nodose ganglia neurons). Overall, there was a modest increase in the size of nodose ganglia neurons after MI. The mean neuronal size in healthy animals was 1204 ± 37 μm^2^ (*n* = 10) versus 1451 ± 61 μm^2^ in LAD-infarcted animals (*n* = 9, *P* = 0.004 vs. healthy animals) and 1411 ± 56 μm^2^ in RCA-infarcted animals (*n* = 7, *P* = 0.014 vs. healthy animals, Supplemental Figure 1; supplemental material available online with this article; https://doi.org/10.1172/jci.insight.155747DS1). No change in soma counts was observed between normal and MI animals.

**Figure 2:**
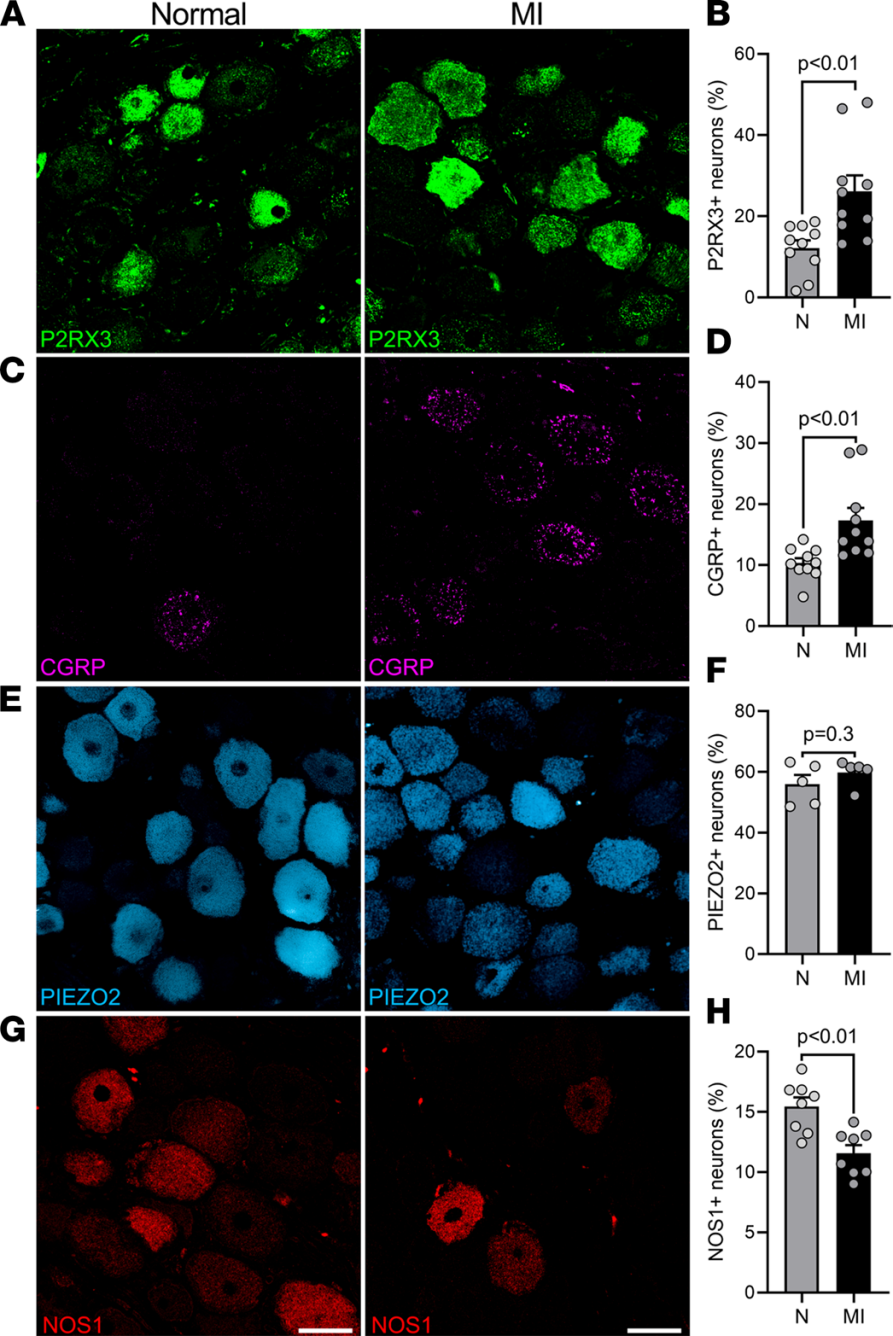
Immunohistochemical assessment of neurons in the porcine nodose ganglia. Representative images of nodose ganglia from normal (left) and LAD-infarcted (right) pig nodose ganglion stained and quantified for (**A** and **B**) P2RX3, (**C** and **D**) CGRP, (**E** and **F**) PIEZO2, and (**G** and **H**) NOS1. Expression of P2RX3 and CGRP were increased after chronic LAD infarction (*P* = 0.005 for normal vs. infarcted animals for P2RX3 and CGRP). PIEZO2 expression remained unchanged (*P* = 0.32). Expression of NOS1 in the nodose ganglia was reduced after LAD infarction (*P* = 0.002 for normal vs. infarcted animals). *n* = 10 pigs per group for P2RX3 and CGRP quantification, *n* = 5 pigs per group for PIEZO2, and *n* = 8 pigs per group for NOS1; data are shown as mean ± SEM; unpaired, 2-tailed Student’s *t* test was used for comparisons. Scale bars are 50 μm.

To assess phenotypic changes associated with MI, expression of receptors and peptides involved in purinergic signaling and cardiac nociception, as reflected by expression of adenosine receptor P2RX3 and CGRP, were assessed. Animals with chronic LAD infarcts exhibited significantly higher expression of both P2RX3 (26.2% ± 3.9% in MI vs. 12.2% ± 1.9% in normal animals, *P* = 0.005) and CGRP (17.3% ± 2.0% in LAD-MI vs. 10.3% ± 0.8% in normal animals, *P* = 0.005), which are receptors and peptides, respectively, involved in nociceptive neurotransmission. Conversely, the expression of the neuromodulator, neuronal NOS1 (11.6% ± 0.7% in MI vs. 15.5% ± 0.7% in normal animals, *P* = 0.002), was attenuated after MI (Figure 2). However, there was no change in the expression of PIEZO2, a mechanosensitive ion channel involved in cardiac mechanotransduction (59.8% ± 1.9% in MI vs. 56.0% ± 3.0% in normal animals, *P* = 0.32, Figure 2) (13). The differences between normal and LAD-infarcted animals remained significant whether data were analyzed by animal (Figure 2) or by individual nodose ganglia (Supplemental Figure 2).

Subsequently, we analyzed the effect of the location of the MI (LAD vs. RCA infarction) on phenotypical changes in nodose ganglia neurons. When comparing neural remodeling between LAD-infarcted (*n* = 10) and RCA-infarcted (*n* = 7) animals, no significant differences in protein expression profiles were noted. Nodose ganglia from RCA-infarcted animals also demonstrated a significant increase in CGRP and P2RX3 expression, a decrease in NOS1 expression, and no significant change in PIEZO2 expression (Supplemental Figure 3), suggesting that these pathological phenotypical changes occurred regardless of the location of infarction. Animals with RCA infarction showed a modestly greater increase in CGRP expression as compared with animals with LAD infarction (27.4% ± 2.7% in RCA- vs. 17.3% ± 2.0% in LAD-MI, *P* = 0.04, Supplemental Figure 3).

### Phenotypical changes in left versus right nodose ganglia.

To assess whether different territories of MI influenced laterality and degree of neural remodeling in the nodose ganglia, left versus right ganglion differences were compared in normal animals and in those with LAD and RCA infarcts. No discernable differences in expression of CGRP, P2RX3, NOS1, or PIEZO2 were observed between the left and right nodose ganglia in normal animals (Figure 1). Similarly, both the left and right nodose ganglia showed comparable pathological changes in LAD- and RCA-infarcted animals, with a lack of laterality in the altered expression of CGRP, P2RX3, NOS1 (Figure 1 and Supplemental Figure 3).

### Basal activity of nodose ganglia neurons in infarcted and healthy animals.

**Figure 3:**
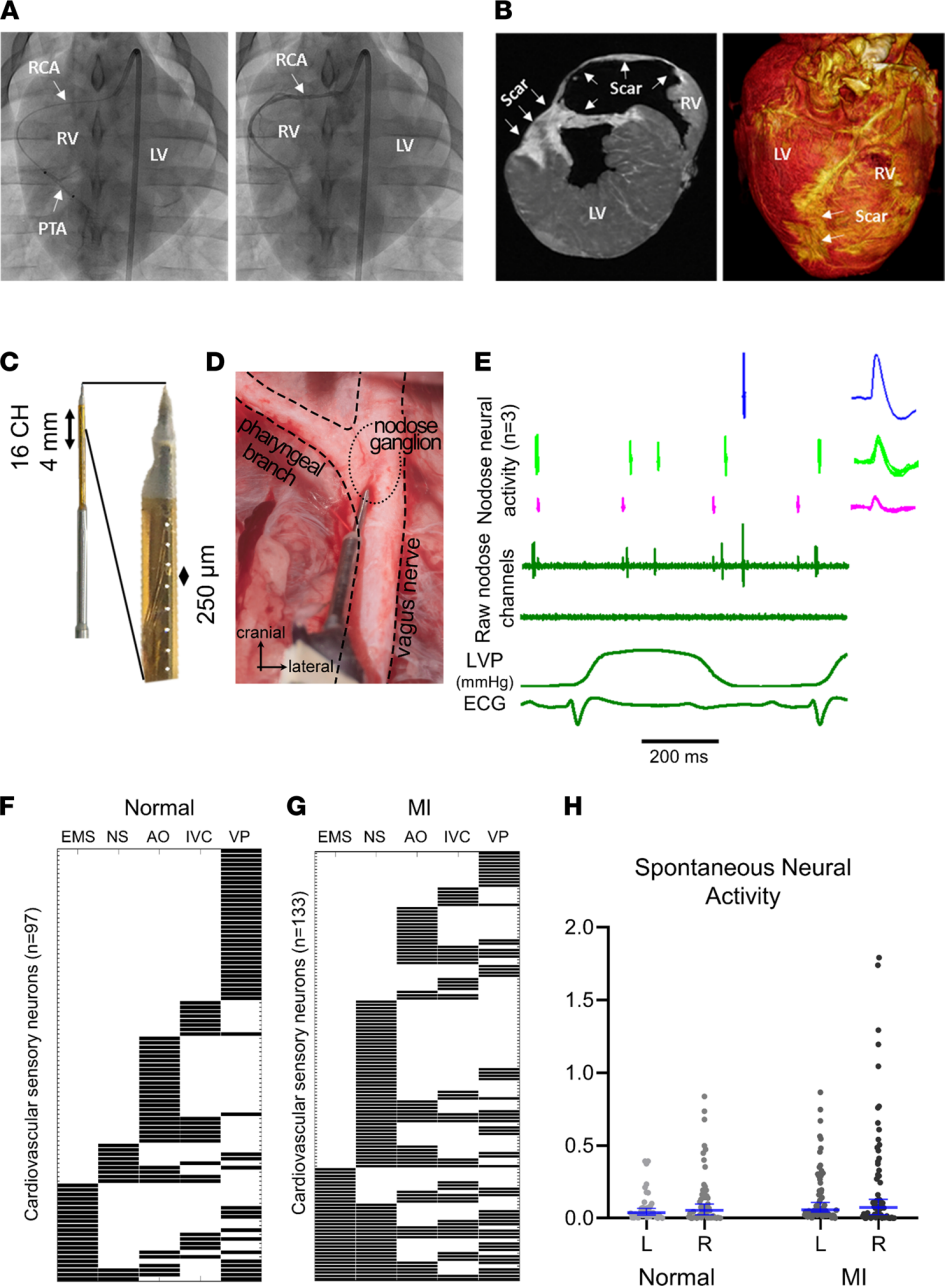
Functional analysis of nodose neuronal activity following MI. (**A**) Creation of right coronary artery myocardial infarct. Left panel: representative coronary angiography (RCA) while placing the percutaneous transluminal angioplasty catheter (PTA) in the RCA. The distal radiopaque marker indicates the distal end of the angioplasty catheters where microspheres are injected. Right panel: Coronary artery angiography after RCA infarct creation. Contrast dye was injected into the RCA to confirm the location of the occlusion. (**B**) Representative magnetic resonance imaging (MRI) images of the RCA-infarcted heart. Scar tissues were confirmed by assessing the thickness and the color of the myocardial regions in the MRI image. (**C**) Customized 16-channel linear microelectrode array probes were used to record nodose neurons in vivo. (**D**) The neural probe was inserted in the nodose ganglion. (**E**) Representative sorted neuronal action potentials for 3 neurons from 1 animal. Action potentials from individual neurons are illustrated in the right panel. Left ventricular pressure (LVP) and electrocardiogram (ECG) were recorded simultaneously with neural recordings. (**F**) Responses of 97 cardiac nodose neurons from 11 normal animals (8.8 ± 2.1 per animal) to epicardial mechanical stimulation (EMS), epicardial nociceptive stimuli (NS), aortic occlusion (AO), inferior vena cava (IVC) occlusion, and ventricular pacing (VP). Only significant responses (*P* < 0.05) to each stressor based on the Skellam test are shown. (**G**) The response of 133 cardiac nodose neurons from 11 animals with MI involving the RCA to cardiac stressors are shown. (**H**) Nodose neuronal firing rate at baseline. There was no difference in the basal activity of nodose neurons in normal versus MI animals. There was no difference in the activity of neurons from the left versus right nodose ganglia. (*n* = 37, 56, 63, and 62 for normal-left nodose, normal-right nodose, MI-left nodose, and MI-right nodose ganglia, respectively.) Median and 95% confidence intervals are shown, and Mann-Whitney *U* statistical test was used for comparisons.

To assess functional changes in cardiac vagal afferent signaling, the activity of neurons in the nodose ganglia in both normal and chronically infarcted animals (chronic RCA infarcts) were recorded in vivo using linear microelectrode arrays (Figure 3). To specifically identify cardiac related nodose neurons, the responses of nodose neurons to cardiac stressors/interventions, including epicardial application of chemical and mechanical stimuli, IVC occlusion, aortic occlusion, and ventricular pacing, were evaluated in all animals (normal: *n* = 11 pigs; RCA-MI: *n* = 11 pigs). Basal neural activity was also analyzed (prior to application of cardiac stressor). Of note, RCA-infarcted animals (which have posterior/dorsal ventricular scars) were used for functional neuronal recording studies to determine responses of vagal afferents to chemical stimuli on the anterior ventricles/viable regions, without the potential confounding effects of scar and denervation that may affect responses. Changes in hemodynamic parameters in response to the above cardiac stressors are shown in Supplemental Table 1. In 11 normal animals, 97 cardiac related neurons (8.8 ± 2.1 per animal), and in 11 infarcted animals, 133 cardiac related neurons (12.1 ± 4.5 per animal), were identified based on their significant responses (using the Skellam test) (14) to cardiovascular interventions (Figure 3).

No significant differences between basal firing rates of left versus right nodose cardiac related neurons in healthy animals were observed (left: 0.04 Hz [IQR 0.01–0.09 Hz], *n* = 37 neurons vs. right: 0.05 Hz [0.004–0.16 Hz], *n* = 56 neurons, *P* = 0.72). Similarly, no differences in the basal firing rates of left versus right nodose cardiac related neurons of infarcted animals were observed (left: 0.06 Hz [0.02–0.27 Hz], *n* = 63 vs. right: 0.07 Hz [0.007–0.38 Hz], *n* = 62, *P* = 0.80) (Figure 3).

Neurons that responded to at least 1 ventricular chemical stimulus and none of the epicardial mechanical stimuli were categorized as chemosensitive cardiac neurons. Mechanosensitive neurons were defined as neurons that responded to epicardial mechanical stimuli and none of the chemical stimuli. If a neuron responded to both mechanical and chemical stimuli, it was defined as a multimodal neuron consistent with previously published criteria (15, 16). The basal activities of mechanosensitive, chemosensitive, and multimodal neurons were compared in normal and infarcted animals to assess any potential differences in basal firing rates of these neurons. No significant difference was found in the basal firing rates of mechanosensitive (MI: 0.12 Hz [0.008–0.49 Hz] vs. normal: 0.16 Hz [0.10–0.38 Hz], *P* = 0.62), chemosensitive (MI: 0.08 Hz [0.03–0.31 Hz] vs. normal: 0.22 Hz [0.08–0.23 Hz] *P* = 0.48), or multimodal (MI: 1.00 Hz [0.32–3.80 Hz] vs. normal: 0.84 Hz [0.74–1.20 Hz] *P* > 0.99) neurons.

### Cardiac phase–related neural activity.

**Figure 4:**
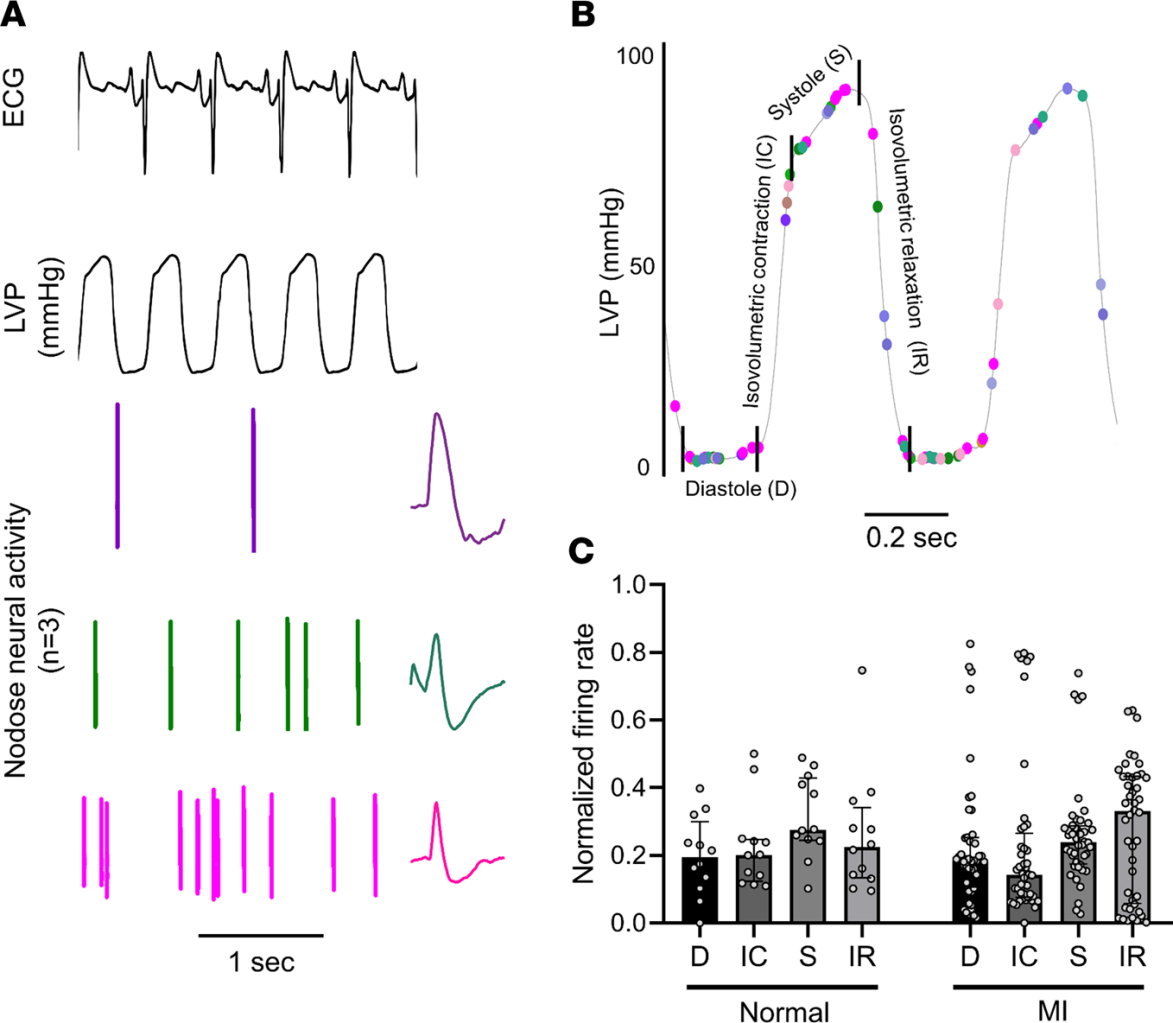
Cardiac phase–related neural activity. (**A**) Representative neural activity from 3 nodose neurons. The first neuron (top, purple) fires during isovolumetric relaxation, the one in the middle fires during systole, and the third (bottom, pink) fires stochastically. The characteristic extracellular action potential for each neuron is also shown on the right. (**B**) Cardiac phases were divided into the following 4 phases: diastole (D), isovolumetric contraction (IC), systole (S), and isovolumetric relaxation (IR). Each dot represents a neural spike, and dots with the same color represent the spiking activity from the same neuron. The position of each dot shows the LVP at each spike time. (**C**) Normalized firing rate of nodose neurons in each cardiac phase. There is no significant difference between normal and RCA-infarcted animals (*n* = 12 for normal and *n* = 44 for MI animals). Median with IQR is shown, and Mann-Whitney *U* statistical test was used.

To assess the preferential activation of nodose neurons during specific parts of the cardiac cycle, the timing of neuronal firing relative to the phase of the left ventricular pressure was evaluated (Figure 4). The cardiac cycle was divided into the following 4 phases: diastole, isovolumetric contraction, systole, and isovolumetric relaxation (Figure 4). Although some nodose neurons showed cardiac phase–related activity in both normal and infarcted animals, there was no significant difference in the normalized firing rates of neurons in each phase of the cardiac cycle between normal and infarcted animals (diastole: 0.19 Hz [0.11–0.30 Hz] for normal vs. 0.18 Hz [0.11–0.25 Hz] for MI animals, *P* = 0.98; isovolumetric contraction: 0.20 Hz [0.12–0.25 Hz] for normal vs. 0.14 Hz [0.08–0.27 Hz] for MI animals, *P* = 0.17; systole: 0.28 Hz [0.24–0.43 Hz] for normal vs. 0.24 Hz [0.17–0.29 Hz] for MI animals, *P* = 0.10; isovolumetric relaxation: 0.22 Hz [0.13–0.34 Hz] for normal vs. 0.33 Hz [0.06–0.43 Hz] for MI animals, *P* = 0.63) (Figure 4), suggesting that mechanotransduction of cardiac cycle pressure changes through the nodose ganglia remained intact after MIs. These findings were consistent with the absence of differences in PIEZO2 channel expression in nodose ganglia of normal versus infarcted animals.

### Neuronal responses to cardiac interventions.

**Figure 5:**
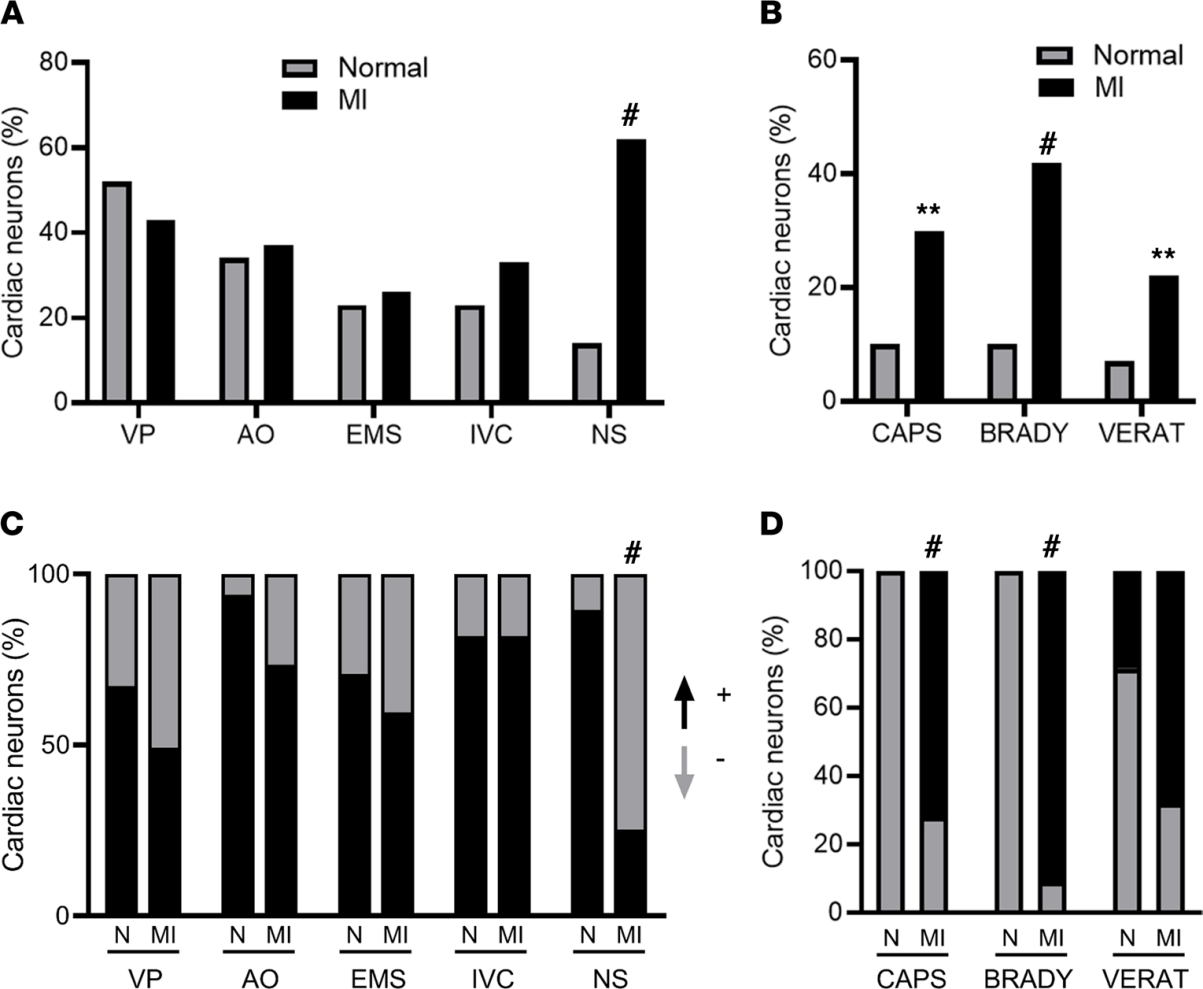
Nodose neural responses to specific cardiac interventions. (**A**) Percentages of neurons with significant changes in absolute firing rates in response to ventricular pacing (VP), aortic occlusion (AO), epicardial mechanical stimulation (EMS), inferior vena cava (IVC) occlusion, and nociceptive stimuli (NS) are shown. (**B**) Percentage of chemosensitive neurons that responded to each specific chemical stimulus is shown (capsaicin, CAPS; bradykinin, BRADY; veratridine, VERAT) in normal and MI animals. (**C**) Percentage of neurons that were excited or inhibited in response to each cardiac stressor is shown. The predominant response to nociceptive stimuli in normal animals was excitatory (an increase in firing rate: +) while the predominant response to nociceptive chemicals in RCA-infarcted animals was inhibitory (a decrease in firing rate: -). (**D**) The percentage of neurons that were excited or inhibited for each specific chemical in normal and RCA-infarcted animals is shown. Both capsaicin and bradykinin caused statistically significant decreases in firing rates. Fisher’s exact test with Dunn’s correction for multiple comparisons was used to compare the percentage of neurons in normal and MI animals. **0.001 < *P* ≤ 0.01, ^#^*P* ≤ 0.001 compared with normal animals.

Chemicals were applied to the anterior/ventral surface of the RV and LV in normal and RCA-infarcted animals. In healthy animals, epicardial application of chemicals (e.g., capsaicin, bradykinin, and veratridine) evoked responses in 13% of neurons, while aortic occlusion, EMS, and IVC occlusion significantly changed firing rates in 34%, 23%, and 23% of neurons, respectively. Ventricular pacing showed the greatest response (52%) in normal animals. In chronically infarcted animals, epicardial application of chemicals engaged the greatest percentage of neurons (60%), followed by ventricular pacing (43%) and aortic occlusion (37%). IVC occlusion activated 33% of cardiac related neurons, and EMS affected the activity of 26% of neurons (Figure 5). When comparing the percentage of neurons responding to specific interventions between infarcted and normal animals, significant differences were only observed in responses to nociceptive chemicals (normal: 13% vs. MI: 60%; Fisher’s exact test, *P* < 0.0001). In keeping with histological findings and cardiac phase synchronization data, no functional differences in responses to LV or RV EMS, or to significant blood pressure changes during IVC and aortic occlusions, in normal versus infarcted animals were observed.

To further evaluate the response of cardiac nodose neurons to nociceptive stimuli, we assessed the percentage of neurons that responded to each nociceptive chemical (Figure 5). Percentages of cardiac sensory neurons that responded to capsaicin (normal: 10% vs. MI: 30%, *P* = 0.01), bradykinin (normal: 10% vs. MI: 42%, *P* < 0.001), and veratridine (normal: 7% vs. MI: 22%, *P* = 0.01) were all increased in infarcted animals.

### Excitatory and inhibitory cardiac sensory responses.

Given the increased prevalence of chemosensitive neurons in infarcted animals, we further probed the magnitude of response and whether nociceptive chemicals had excitatory or inhibitory effects on cardiac vagal neurotransmission, as these responses after MI were unknown. In general, most cardiac stressors caused both excitatory and inhibitory effects on the activity of cardiac sensory neurons (Figure 5). In response to nociceptive stimuli, nodose neurons in healthy animals showed a higher incidence of excitatory responses (increase in firing rates). Although the magnitude of the absolute firing rates (regardless of activation or inhibition) was higher in response to nociceptive chemicals after MI (Supplemental Figure 4), surprisingly, the application of nociceptive chemicals in infarcted animals showed a predominantly inhibitory response (Figure 5). Unlike normal animals, the majority of the neurons in the MI animals decreased their firing rates in response to a chemical application (normal: 90% excitatory, 10% inhibitory vs. MI: 25% excitatory, 75% inhibitory, *P* < 0.001). No differences in excitatory or inhibitory responses were observed for other cardiac interventions, including aortic and IVC occlusions, where interventions caused similar excitatory and inhibitory responses in MI versus normal animals.

**Figure 6:**
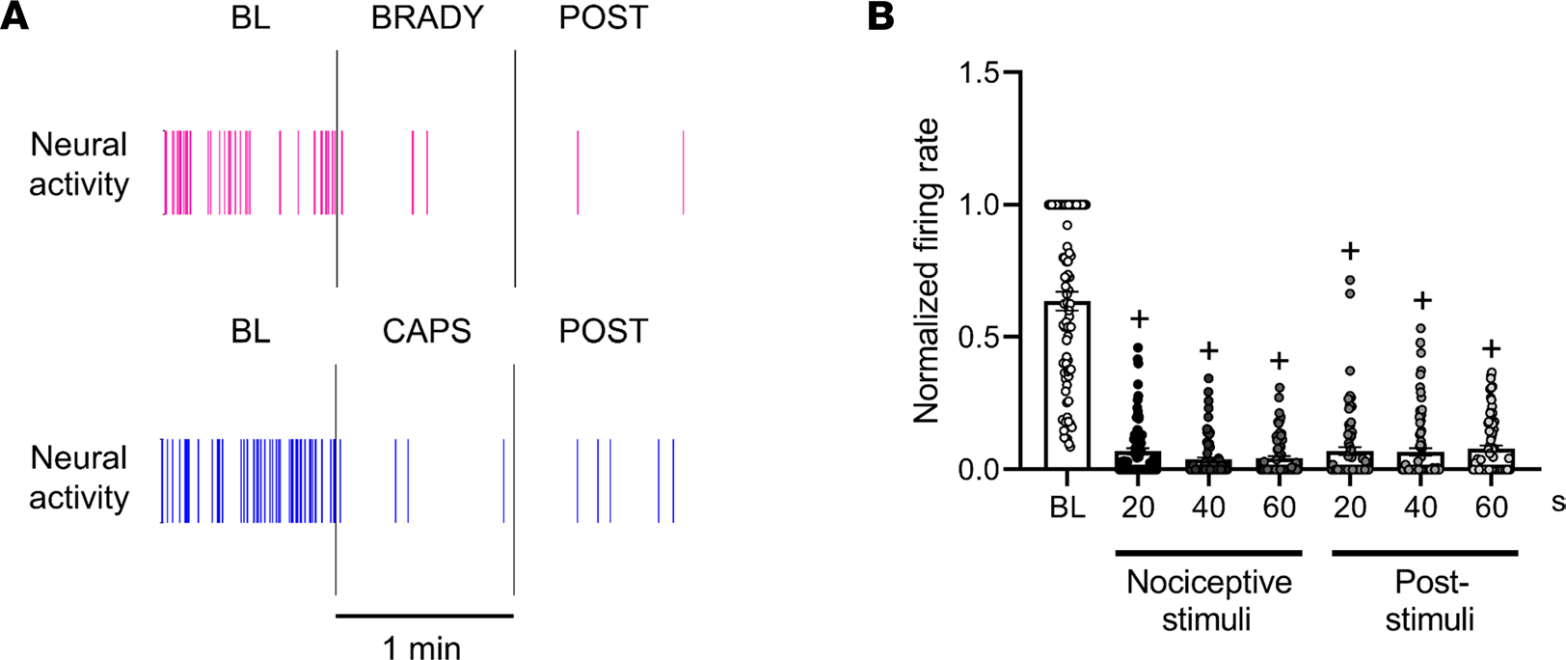
Temporal profile of nociceptive neural responses in infarcted animals demonstrates inhibition of firing during nociceptive chemical application of capsaicin and bradykinin, which persists after removal of the nociceptive chemical. (**A**) Representative inhibitory response of 2 nodose neurons to bradykinin (BRADY) and capsaicin (CAPS) in a chronic RCA infarct. (**B**) Quantified temporal inhibitory responses of neurons (*n* = 80) to nociceptive stimuli in the RCA-infarcted animals show persistence of inhibition of neural firing rates even after removal of the stimulus/chemical. Data are shown as mean ± SEM. Dunn’s multiple-comparison tests were used to compare the normalized firing rate versus baseline (BL). ^+^*P* ≤ 0.001 compared with BL.

Since the responses to cardiac nociceptive stimuli seemed to be specifically affected after MI, additional detailed analyses of neural responses to epicardial application of each nociceptive chemical were undertaken. In normal animals, capsaicin and bradykinin both evoked purely excitatory responses from all recorded chemosensitive nociceptive neurons (i.e., an increase in firing rate was seen in these neurons in response to bradykinin and capsaicin, Figure 5). Surprisingly, in chronically infarcted animals, capsaicin evoked a significant decrease in the firing activity of 91% of recorded chemosensitive/nociceptive neurons, while bradykinin resulted in an inhibitory response in 72% of chemosensitive/nociceptive neurons (*P* < 0.001 vs. responses in normal animals for bradykinin and capsaicin). Furthermore, cardiac sensory neurons that were inhibited by 1-minute application of nociceptive chemicals showed continued inhibition of firing compared with baseline for up to 1 minute after removal of the chemical and cessation of stimuli (Figure 6). In normal animals, the temporal response of cardiac neurons that showed excitatory responses to nociceptive stimuli terminated after removal of the nociceptive stimulus (Supplemental Figure 5).

### Modulation of nociceptive neurons via GABAergic expression, glial activation, and nitric oxide signaling.

**Figure 7:**
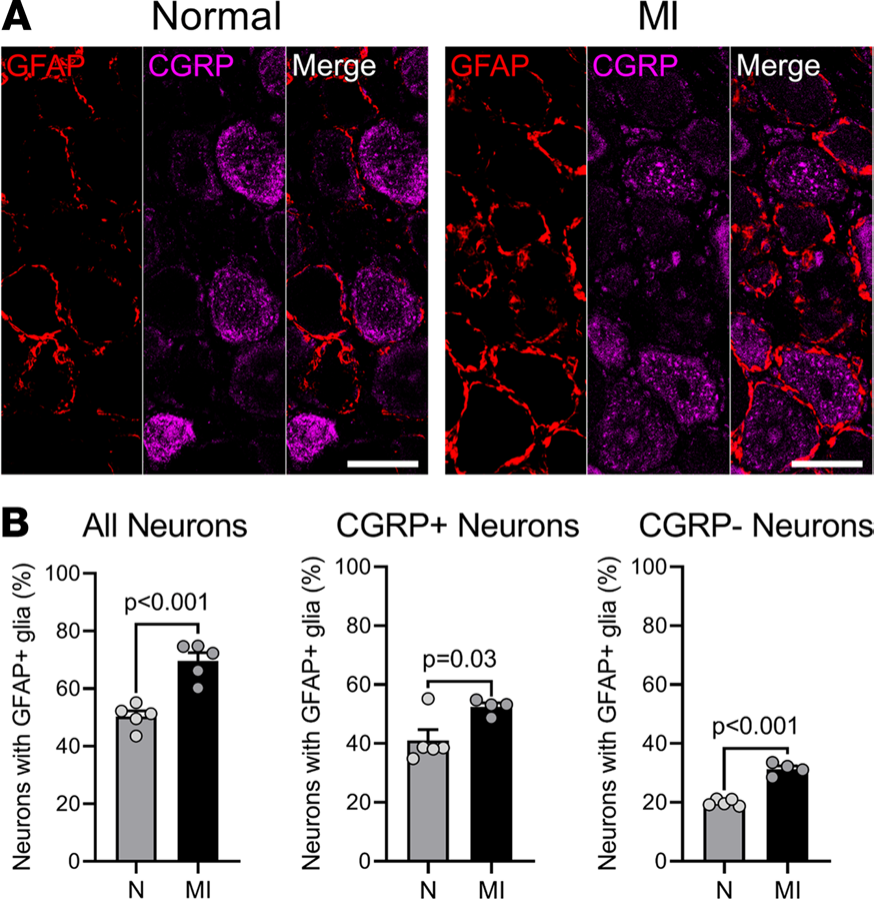
Nonselective augmentation of satellite glial cell activation following MI in the nodose ganglia. (**A**) Representative images of nodose ganglia from normal (N; left) and LAD-infarcted (MI; right) pigs stained for GFAP (red) and CGRP (purple). (**B**) Summary of the percentage of neurons surrounded by GFAP^+^ satellite glial cells as a subset of all neurons, CGRP^+^ neurons, and CGRP^–^ neurons is shown. There was a statistically significant increase in GFAP expression after MI (*P* < 0.001 for infarcted vs. normal animals). This was, however, true for both CGRP-positive (*P* = 0.03 for infarcted vs. normal animals) and CGRP-negative neurons (*P* < 0.001 for infarcted vs. normal animals). *n* = 5 pigs per group in all neurons. *n* = 5 normal pigs and *n* = 4 for MI animals for coexpression. Data are shown as mean ± SEM; unpaired, 2-tailed Student’s *t* test was used for comparisons. Scale bars are 50 μm.

To determine potential alterations in neuronal phenotype that may explain why nodose nociceptive neurons from chronically infarcted animals may display an inhibitory response, we analyzed additional neurochemical changes in CGRP-positive neurons, as these neurons are involved in nociception and release CGRP upon activation of transient receptor potential cation channel subfamily V member 1 (TRPV1). Several potential neuromodulatory factors have been shown to affect the activity of nociceptive neurons in other sensory ganglia (i.e., dorsal root ganglia). These include glial activation, changes in GABA expression, and reduction in NOS1 (17–19). We first determined the expression of glial fibrillary acidic protein (GFAP) in nodose ganglia and then its expression in satellite glia cells that encircled CGRP-expressing nodose neurons in normal versus infarcted animals. GFAP expression was significantly increased in the ganglia from infarcted animals (MI animals 69.6% ± 2.8% vs. 50.3% ± 1.9% in normal animals, *P* < 0.001), but these differences were observed for both CGRP-expressing and CGRP-negative neurons, suggesting a more global phenotype after MI (Figure 7 and Supplemental Figure 6). Given that nociceptive signaling was largely altered after MI, the global changes in observed GFAP expression, thus, were unlikely to be a primary reason for alterations in nociceptive neurotransmission.

**Figure 8:**
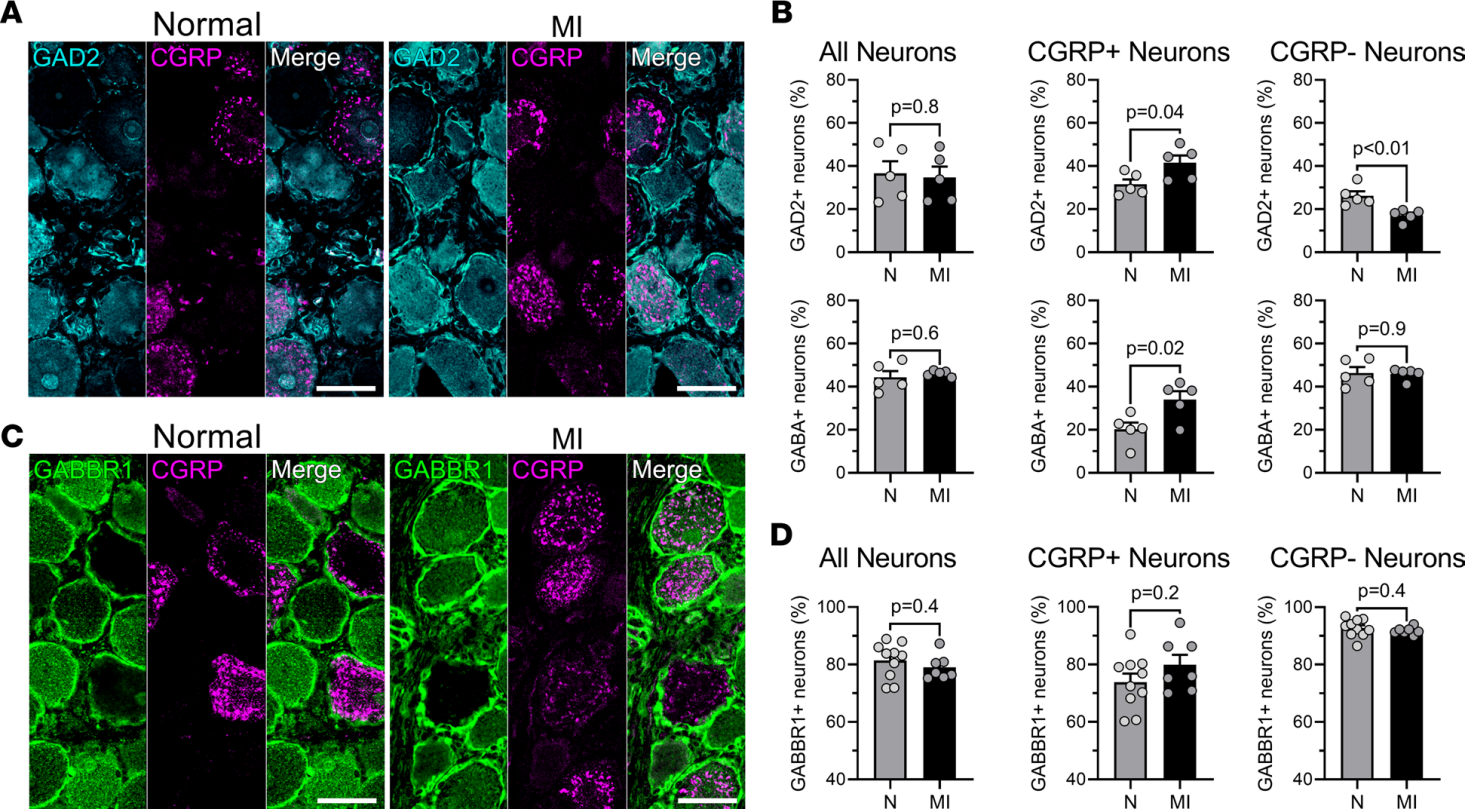
Upregulation of inhibitory GABAergic neurotransmission specifically in CGRP-expressing nodose ganglia neurons involved in nociceptive neurotransmission. (**A**) Representative images of nodose ganglia from normal (N; left) and LAD-infarcted (MI; right) animals stained for GAD2 (blue) and CGRP (purple). (**B**) *Top,* summary of the percentage of GAD2^+^ neurons as a subset of all neurons, CGRP^+^ neurons, and CGRP^–^ neurons is shown. Although overall expression of GAD2 was unchanged between normal and LAD-infarcted animals (*P* = 0.80), the percentage of neurons that coexpress both GAD2 and CGRP was increased after MI (*P* = 0.04 for LAD-infarcted vs. normal animals). Coexpression of GAD2 was decreased in neurons that do not express CGRP (*P* = 0.006 for normal vs. LAD-infarcted animals). *n* = 5 pigs per group. *Bottom,* summary of the percentage of GABA^+^ neurons as a subset of all neurons, CGRP^+^ neurons, and CGRP^–^ neurons is shown. Similar to GAD2, GABA showed no overall difference (*P* = 0.57) while its expression in CGRP^+^ neurons was increased (*P* = 0.02) in MI animals. However, expression of GABA in CGRP^–^ neurons was similar between normal and MI animals (*P* = 0.89). (**C**) Representative images of nodose ganglia from normal (left) and LAD-infarcted (right) pigs stained for GABA type B receptor subunit 1 (GABBR1) (green) and CGRP (purple). There was no difference in the expression of GABBR1. *n* = 5 pigs per group. (**D**) Summary of the percentage of CGRP^+^ neurons with GABBR1^+^ expression (*P* = 0.21). *n* = 10 for normal and *n* = 7 for MI animals. Data are shown as mean ± SEM; unpaired, 2-tailed Student’s *t* test. Scale bars are 50 μm.

GABA is another known neuromodulator in peripheral sensory autonomic ganglia seen to be expressed by neurons of the trigeminal, nodose, and dorsal root ganglia (DRG) (19, 20). In DRG, GABA has been reported to produce a net inhibitory effect on peripheral nociceptive neurotransmission (19). In nodose neurons, these effects of GABA have further been shown to be nonsynaptically mediated, spreading between neurons and glia (21). Given previous findings that GABA may perhaps induce global vagal silencing and our in vivo functional findings, changes in GABAergic expression in the nodose ganglia after MI were evaluated. No differences in expression of GABA or glutamic acid decarboxylase (GAD2), the enzyme required for GABA synthesis, were noted in the nodose ganglion globally (Figure 8 and Supplemental Figure 7). However, a significant increase in the percentage of CGRP-positive neurons that coexpress GABA (34.0% ± 3.9% in MI vs. 20.2% ± 3.1% in normal, *P* = 0.02) and GAD2 was observed (41.6% ± 3.4% in MI vs. 31.6% ± 2.3% in normal, *P* = 0.04) after MI. Interestingly, CGRP-negative neurons demonstrated decreased expression of GAD2 (17.2% ± 1.2% in MI vs. 26.2% ± 2.1% in normal, *P* = 0.006) in infarcted compared with normal animals. suggesting that GABA may be a factor in the greater inhibitory responses observed to nociceptive chemicals. In addition, as the presence of GABA type B receptors, and especially the GABBR1 subunit, had not been previously described in the nodose ganglia of larger mammals to our knowledge, we confirmed the presence of GABBR1 in these ganglia and found that approximately 70%–80% of CGRP-expressing neurons also expressed GABBR1 in both normal and infarcted pigs (Figure 8 and Supplemental Figure 7).

Finally, NOS1, another known autonomic and afferent neuromodulator, was globally decreased following MI (Figure 1), and we evaluated whether these alterations were specific to CGRP-expressing neurons and may explain the differences in observed functional responses. Despite an overall decrease in NOS1 expression after MI, nociceptive CGRP neurons were not selectively affected (19.7% ± 1.6% in normal vs. 25.5% ± 2.4% in MI; *P* = 0.07, Supplemental Figure 8).

## Discussion

To our knowledge, this is the first study to evaluate the effects of chronic MI on vagal afferent neurotransmission using both functional (direct in vivo neural recordings) and immunohistochemical data. A major finding of this study is that, while the percentage of nociceptive neurons was increased after MI, as assessed by histological and functional responses, the predominant response observed in infarcted animals was significant inhibition (*P* < 0.01) of neural activity with application of nociceptive chemicals. This was significantly different from normal animals, in whom the predominant response was excitatory. This potentially novel finding has important implications for vagal dysfunction. In addition, for the first time to our knowledge, we show that both LAD and RCA myocardial infarcts caused pathological changes in the expression profiles of neurons of the right and left nodose ganglia. No differences between the right versus left nodose ganglia (i.e., no laterality) were observed with respect to neuronal receptors, peptides, or functional responses to cardiac interventions, suggesting that infarction of a particular region of the heart has global effects on both ganglia. This study also demonstrates that the proportion of PIEZO2-expressing neurons was unchanged after MI; consistent with these findings, the functional neuronal responses to EMS, IVC occlusion, and aortic occlusion were comparable between normal and infarcted animals. Similarly, specific differences were not found in cardiac phase–related neuronal activity between infarcted and normal pigs. Finally, the percentage of CGRP-positive neurons that express GABA was significantly increased (*P* = 0.02) after MI, which may suggest that GABA may play a role in the functional differences in nociception observed after MI.

The autonomic nervous system controls every aspect of cardiac function. Sensory pseudounipolar neurons in DRG and nodose ganglia contain a diverse population of chemosensitive and mechanosensitive neurons that sense the cardiac milieu and transmit this information to the spinal cord and brainstem, respectively (1, 5, 22–24). Activation of nociceptive neurons in DRG increases sympathetic tone, while activation of nociceptive neurons in nodose ganglia increases the central vagal drive to the heart (25–28). Therefore, the effects of cardiac nociceptive stimuli in normal animals are determined by the balance between sympathetic and vagal output. It is established that chronic MI leads to reduced vagal drive, increasing the risk of ventricular arrhythmias and heart failure. Augmenting vagal drive via vagal nerve stimulation has been shown to be antiarrhythmic in animal models (5, 6, 29, 30). Yet, mechanisms behind central vagal dysfunction after MI are unknown. We hypothesized that MI, as a form of chronic injury, similar to chronic pain conditions that cause remodeling in DRG, may cause alterations in nodose ganglia signaling that then drive reduced vagal function. Toward this goal, an investigation was undertaken in a large-animal (porcine) model to assess both structural and functional changes after chronic MI. This animal model has been established to undergo neural, electrical, and cardiac remodeling that is very similar to humans after MI (5, 31, 32).

Different markers for mechano- and chemotransduction were utilized in this study. PIEZO2 receptors on neurons in the nodose ganglia are thought to sense visceral and cardiovascular mechanical stretch (33, 34), and expression of PIEZO2 was used as a marker for mechanosensitive neurons. In this study, no histological differences or functional changes, as assessed by cardiac phase–related activity or transduction of pressure-associated interventions, in mechanosensitivity of vagal afferents were observed. Of note, in our model of MI, hypertension and significant LV hypertrophy do not occur, and it is possible that in the setting of hypertensive heart disease, PIEZO2-sensitive neurons in the nodose ganglia may remodel, as suggested by a mouse model of aortic constriction (35).

In this study, in healthy animals, nociceptive stimuli engaged the lowest number of neurons (10% of all identified cardiac neurons), suggesting a high threshold for activation of these neurons in the setting of a normal heart. The responses observed were predominantly excitatory. In animals after MI, however, the same application of nociceptive chemicals affected the activity of a greater proportion (48%) of all cardiac neurons, and the predominant responses observed were inhibitory. It has also been previously shown that acute application of nociceptive chemicals, such as capsaicin, bradykinin (36, 37), and veratridine (38), causes neuronal depolarization and predominantly increases in activity and firing rates of the majority of nociceptive neurons in the peripheral autonomic ganglia when evaluated either in vivo or using whole-cell voltage-clamp recordings (39, 40). In vivo nodose recordings from guinea pigs with healthy hearts, which were consistent with the healthy porcine animal data in this study, showed that epicardial application of bradykinin activated the majority (74%) of identified cardiac nociceptive neurons, with a decrease in firing rate observed in only 26% of recorded neurons (22). Unexpectedly, neuronal analyses of cardiac sensory nodose neuronal activity after chronic MI showed that application of nociceptive chemicals caused a predominantly inhibitory response in the vast majority of recorded chemosensitive neurons. For all other cardiac stressors except nociceptive stimuli, the percentages of inhibitory and excitatory responses were not different in normal versus infarcted animals. This was despite the application of these chemicals to viable, noninfarcted regions of the heart and has significant implications for vagal neurotransmission, given that excitatory responses by nociceptive chemicals are known to increase vagal tone. For example, a nociceptive stimulus, such as a second ischemic insult, in an already diseased heart would lead to a reduction in vagal tone (as opposed to an increase), further increasing the risk of ventricular arrhythmias. Although the effect of ischemia was not assessed in this study, given the complex responses to ischemia that involve activation of both mechano- and chemosensitive neurons, the neuropeptides and receptors evaluated, including CGRP and P2RX3, are known to be released and activated during ischemia, respectively (41). In addition to ischemia-induced release of ATP, which activates P2RX3 receptors, release of CGRP is known to occur with activation of TRPV1 receptors because of the low pH ischemia causes. Finally, ischemia also leads to release of bradykinin (42). Thus, it is reasonable to assume that given altered responses to nociceptive chemicals, vagal afferent responses due to ischemia would also be altered in infarcted animals.

The reason for the inhibitory neural response observed in chronic infarcted animals requires further investigation, but greater expression of GABA may play a role (19, 20, 43, 44). GABA is known to reduce nociceptive neurotransmission through DRG, where activation of TRPV1 nociceptive neurons releases not only CGRP but also GABA upon binding of chemicals such as capsaicin (45). GABA was reported to act on TRPV1-coupled GABBR1 of these neurons, reducing further activation, not only of the same neuron but also of nearby nociceptive neurons, in a negative feedback fashion (43). In this study, a greater percentage of CGRP-positive neurons in the nodose after MI expressed GABA compared with controls. Therefore, it is possible that remodeling of nodose neurons after chronic MI that leads to greater expression and release of GABA by CGRP-expressing nodose neurons may inhibit surrounding TRPV1 receptors/CGRP-positive neurons, resulting in more inhibitory functional responses (19). In this study, we further show that nodose neurons also express the GABBR1, with a large percentage (approximately 70%–80%) of CGRP-expressing neurons coexpressing GABBR1 in this large-animal model.

In this study, we investigated several other immunohistochemical changes that are thought to modulate peripheral neurotransmission to see if they were responsible for functional changes that were observed in nociceptive neurons. Activation of satellite glial cells in response to injury, as reflected by increased GFAP (a reactive cytoskeletal protein), has been shown to modulate neuronal excitability and function in other peripheral ganglia (17, 46). In this study, although an increase in GFAP globally in the nodose ganglia after MI was also observed, this increase was not selective for CGRP-expressing neurons. Finally, nitric oxide is an important neuromodulatory molecule, and a decrease in neuronal nitric oxide synthase can alter neuronal signaling (47). Nitric oxide in the nodose ganglia is thought to enhance cholinergic neurotransmission (18, 48) and improve baroreceptor sensitivity (49). Reduction in release of NOS1, therefore, could potentially decrease sensory afferent transduction. However, NOS1 expression, although reduced globally, did not selectively affect CGRP-expressing neurons and tended to affect CGRP-negative neurons.

In summary, this is the first study to our knowledge to show that chronic MI causes both structural and functional changes in vagal afferent nociceptive signaling that can exacerbate parasympathetic dysfunction and reduce vagal tone. Responses to nociceptive stimuli were overall inhibitory, rather than excitatory, a change that may be mediated by increased GABAergic expression in nociceptive neurons. Inhibitory responses in vagal afferent signaling would result in decreased central vagal efferent drive and may explain why patients after MI experience parasympathetic dysfunction, increased risk of ventricular arrhythmias, and associated sudden death. Future studies following up on specific alterations reported herein will likely shed further light on the effects of MI on the activity of specific types of vagal neurons and modulation of a specific pathway.

Our study has limitations. General anesthesia is known to suppress neuronal activity. In this study, after completion of surgical procedures, isoflurane was discontinued and α-chloralose used as an alternative anesthetic agent to minimize the effects of isoflurane. Nevertheless, it is possible that the neuronal responses in this study are a conservative estimate of those that would be observed in conscious animals. For neural recordings, an RCA infarct model was used in order to allow for epicardial application of mechanical/chemical stimuli on the viable, accessible ventral regions of the ventricles. It is possible that the application of chemicals to infarcted regions would lead to different responses. Given the lack of previous data on post-MI alterations in nodose signaling, viable regions were studied in order to avoid effects that may be caused by denervation and fibrosis. Acute effects of stimuli were evaluated. Cardiac neural responses to chronic stimuli may be different due to neuronal memory or remodeling of receptors. Direct staining for TRPV1 expression was not performed, given the lack of specific antibodies in the porcine model, and CGRP expression was used as a surrogate for TRPV1. It is, however, established that the large majority of TRPV1-expressing neurons express and release CGRP (50, 51). In this study, assessment of efferent vagal tone using direct vagal efferent nerve recordings was not performed, and given the short duration (1 minute) of interventions, heart rate variability analysis during stimuli was not appropriate. However, using the same infarct model and direct intrinsic cardiac neural recordings from postganglionic intrinsic cardiac parasympathetic neurons, our laboratory has shown previously that central vagal tone and inputs to intrinsic cardiac ganglia neurons are altered, consistent with decreased central vagal drive (5). In a canine infarct model, lower central vagal drive during ischemia is associated with the occurrence of ventricular fibrillation (52). Finally, direct application of GABA agonists or antagonists was not performed to confirm restoration of nociceptive neurotransmission in infarcted animals. Therefore, although the findings of this study are suggestive, the role of GABA in nociceptive neurotransmission within the vagal ganglia requires confirmation in future studies.

## Methods

Yorkshire pigs (Premier BioSource) were used for histological evaluation and in vivo nodose neural recording. For histological assessment, normal Yorkshire pigs (*n* = 22, 52.1 ± 2.5 kg, 20 males) and Yorkshire pigs with healed anterior/apical (ventral) MI involving the LAD (*n* = 24, 58.6 ± 2.9 kg, 22 males) or inferior/dorsal MI involving the RCA (*n* = 7, 56.9 ± 3.0 kg, 7 males) were used (Supplemental Table 2). For extracellular nodose neural recordings, normal Yorkshire pigs (*n* = 11, 58.2 ± 3.2 kg, 9 males) and Yorkshire pigs with healed RCA myocardial infarcts (*n* = 11, 59.5 ± 1.7 kg, 9 males) were used.

Creation of myocardial infarcts

Percutaneous MI in the region of the RCA was created in 42 pigs (30–35 kg) as previously described (5, 12). Briefly, animals were sedated (tiletamine-zolazepam, 4–8 mg/kg IM), intubated, and placed under general anesthesia with isoflurane (1%–5% inhaled). For RCA infarcts, under fluoroscopic guidance, an 8F AL2 guide catheter (Boston Scientific) was advanced from the femoral artery to the ostium of the RCA. A 2.5 to 3.5 mm percutaneous transluminal angioplasty balloon catheter (Abbott) was advanced over a balance middleweight universal guidewire (Abbott) into the RCA and positioned after the takeoff of the atrioventricular nodal artery (Figure 1). The balloon was inflated, and 3–5 mL of polystyrene microspheres (Polybead 90.0 μm, Polysciences) followed by 5 mL normal saline were injected through the lumen of the catheter. The balloon was then deflated and removed. MI was confirmed by the presence of ST elevation or T wave inversion on ECG and coronary angiography showing a lack of flow in the artery (Figure 1). After the procedure, ECG and blood pressure were monitored for 20 minutes before extubation. Immediate external defibrillation was performed if the animal developed VT or fibrillation. After extubation, animals were monitored until they could ambulate.

For LAD infarcts, a similar procedure was performed, but the AL2 was used to cannulate the ostium of the left main coronary artery and a balance middleweight universal wire advanced into the LAD, instead of the RCA. The percutaneous transluminal angioplasty balloon catheter was inflated after the first diagonal branch, followed by microsphere injection (5, 12). Six to 8 weeks were allowed for maturation of MI in all infarcted animals prior to terminal studies.

### Immunohistochemical analysis

Animals were sedated, intubated, and placed under anesthesia as described above. Lateral neck dissections were performed, and the vagus nerve was isolated bilaterally. The nerve was followed superiorly, and the inferior vagal (nodose) ganglia were identified (Figure 1). Bilateral nodose ganglia from infarcted animals 6–8 weeks post-MI and from normal animals of similar weight were removed. Ganglia were fixed in 4% paraformaldehyde for 24 hours at 4°C and then embedded in paraffin. Tissue was sectioned (5 μm) longitudinally, and midsections of each ganglion with the largest cross-sectional area were used for staining. Sections were deparaffinized in 2 xylene washes followed by rehydration in 3 ethanol washes and water. Antigen retrieval was performed using EDTA buffer, pH 8.0 (90°C for 20 minutes; ab64216; Abcam). Slides were blocked for 1 hour in 3% BSA-TBS-0.2% Triton X-100 with 5% donkey serum and incubated overnight at 4 °C with primary antibody (Supplemental Table 2). Slides were subsequently incubated for 1 hour at room temperature with the appropriate affinity-purified F(ab′)_2_ fragment secondary antibody (Supplemental Table 2). Subsequent steps varied depending on the method of detection. For immunofluorescence, slides were counterstained and mounted using Vectashield with DAPI (Vector Laboratories; H-1200). Slides were imaged at ×100 original magnification (×10 objective, ×10 eyepiece) using a Zeiss LSM 880 with Airyscan. For bright-field detection, the avidin-biotin complex method (PK-6100; Vector Laboratories) of signal amplification followed by 3,3′-diaminobenzidine to induce chromogenic color development was used. Slides were then counterstained with Harris’s hematoxylin, dehydrated, and mounted with Permount (Fisher Chemical, Thermo Fisher Scientific; SP15-100). Slides were imaged at ×400 magnification (×40 objective, ×10 eyepiece) using an Aperio ScanScope AT (Leica Biosystems). The number of nodose neurons or glia expressing a particular antigen was quantified (NIH ImageJ 1.8.0) by 2 independent researchers. Data from left and right nodose were analyzed both by individual ganglia and by animal. All scale bars shown in figures are 50 μm.

### Surgical preparation for neural recording experiments

Animals were sedated, intubated, and placed under anesthesia as described above. Isoflurane was used for surgical procedures/sternotomy and transitioned to α-chloralose (MilliporeSigma; 50 mg/kg initial bolus, after that 20–35 mg/kg/h IV) for the neural recording portion of experiments. Depth of anesthesia was adjusted based on corneal reflex, jaw tone, and hemodynamics indices. Peripheral capillary oxygen saturation (SpO_2_), end-tidal CO_2_, and arterial blood gases were monitored throughout the experiments; tidal volume and/or respiratory rate were adjusted or sodium bicarbonate (HCO_3_) was administered to maintain a normal pH. Body temperature was monitored and adjusted using a water heating pad (Gaymar T/Pump, Gaymar Inc.). The CardioLab System (GE Healthcare) was used to record continuous 12-lead ECGs. Ventral precordial leads were placed posteriorly given sternotomy. The femoral and carotid arteries were cannulated to measure blood pressure and obtain access to the LV for pressure recordings, respectively. Sheaths were placed in the femoral veins for delivery of medications and saline. ECG, arterial pressure, and LVP were digitized (Power 1401, Cambridge Electronic Design); stored; and analyzed off-line (Spike2, Cambridge Electronic Design). Fentanyl boluses (20–30 mcg/kg) were used during sternotomy to reduce discomfort. Sodium pentobarbital (Med-Pharmex Inc.; 100 mg/kg IV) followed by saturated KCl (MilliporeSigma; 1–2 mg/kg IV) was used for euthanasia.

### Nodose neuronal activity recordings

For in vivo extracellular neural recordings of the nodose ganglia, vagi were kept intact during neural recordings. Custom-made 16-channel linear microelectrode arrays (MicroProbes; 25 μm diameter platinum/iridium electrodes, 16 electrodes/probe, 250 μm interelectrode spacing) were gently advanced into the nodose ganglia and connected to a 16-channel preamplifier (NeuroNexus). Neural signals were sampled at 20 kHz, amplified, and digitized (SmartBox acquisition system, NeuroNexus). The signals were filtered (300 Hz to 10 kHz). In vivo soma activity of individual neurons was assessed at baseline and during each cardiovascular intervention. Activity from axons of passage was not recorded, as with high-impedance neural probes, it is not possible to record axonal action potentials (10). Linear microelectrode array position was adjusted if no cardiac related neuronal activity was observed.

### Cardiovascular interventions

To characterize and identify cardiovascular nodose neurons, the following cardiovascular stimuli were applied: ventricular epicardial i) mechanical and ii) nociceptive (capsaicin, bradykinin, veratridine) stimuli, iii) ventricular pacing, iv) IVC occlusion, and v) descending aortic occlusion. Interventions were performed in a random order in all animals; however, application of chemical stimuli was performed last, and in particular, the application of capsaicin was performed as the last intervention in each experiment, given previous data suggesting that capsaicin may alter afferent transduction to subsequent stimuli. A 15- to 30-minute waiting period was allowed for hemodynamic indices to return to baseline between interventions.

#### Ventricular EMS.

To identify nodose ganglia neurons that respond to mechanical stimuli, gentle pressure (~10 g/cm^2^) was applied to the right and left anterior ventricular epicardium for 15 seconds via a saline-soaked cotton-tipped applicator.

#### Ventricular epicardial nociceptive stimuli.

Nociceptive neurons were identified by assessing the response of nodose neurons to a 1-minute application of gauze soaked in bradykinin (1.06 mg/mL), capsaicin (0.03 mg/mL), or veratridine (0.67 mg/mL), placed on the ventral (anterior) aspect of the left and right ventricular epicardium.

#### Ventricular pacing.

The right ventricle was paced endocardially for 1 minute at 15% above the baseline heart rate and intermittently every 4–7 beats with random/variable coupling intervals (300–600 ms) via a quadripolar catheter (Abbott) placed from the femoral vein and attached to a cardiac stimulator (EPS320, MicroPace).

#### Great vessel occlusions.

The IVC and then descending aorta were occluded for 30 seconds via 2 umbilical tapes placed around the IVC and descending aorta. Afferent neural responses to changes in preload and afterload were evaluated.

### Neural signal processing and analysis

Neural signals were processed using Spike2 software (Cambridge Electronic Design) (5, 15, 16). Artifacts were identified and removed by detecting similar and simultaneous waveforms on all neural channels. Neural spikes were identified using a threshold of 3× signal-to-noise ratio. Spike classification was performed using principal component, cluster on measurements, and K-means clustering analysis. The spiking activity of each individual neuron was obtained for the entire experiment and transferred to MATLAB (MathWorks) for post hoc neural analysis.

Neuronal responses to each intervention were assessed by comparing the neural activity at baseline (1 minute) to “during the intervention” using the Skellam statistical test (14). Cardiac related neurons were identified as those that significantly responded to at least 1 cardiovascular stressor. All non–cardiac related neurons were excluded from post hoc analysis. Neurons that responded to at least 1 ventricular epicardial chemical stimuli and none of the epicardial mechanical stimuli were labeled as chemosensitive cardiac neurons. Neurons that responded to nociceptive chemicals (capsaicin, bradykinin, and veratridine) were classified as nociceptive chemosensitive neurons. Mechanosensitive neurons were defined as neurons that responded to the epicardial mechanical stimuli and none of the chemical/nociceptive stimuli. If a neuron responded to both ventricular epicardial mechanical and chemical stimuli, it was classified as a multimodal neuron.

### Data availability

Data supporting this study are available from figshare, https://figshare.com/s/668393bb2dbe87d159b6, and from the corresponding author upon reasonable request.

### Statistics

Neuronal responses to interventions were assessed by Skellam’s significant test, which has been validated for neural activity in nodose and other peripheral ganglia (5, 15, 16), as well as the central nervous system (14). Neural responses to different cardiovascular stressors were compared using Fisher’s exact test. Anderson-Darling test was used to check data for normality of distribution. Paired data were compared using 2-tailed Student’s paired *t* test or Wilcoxon’s signed-rank test, depending on the distribution. Unpaired data (normal vs. MI) were compared using unpaired *t* test or Mann-Whitney *U* test, depending on Gaussian distribution. For histological data, comparison of molecular expression profiles between normal and infarcted animals was performed using unpaired, 2-tailed Student’s *t* test. Phenotypic comparisons within an animal (e.g., left vs. right) were performed using paired, 2-tailed Student’s *t* test. Benjamini-Hochberg method was used to correct for the false discovery rate. Dunn’s multiple-comparison test was used to compare neural firing rates at baseline with multiple time points. Prism (GraphPad) was used for all statistical analyses. Data are presented as mean ± SEM or median and IQR [25th, 75th percentiles]. A *P* ≤ 0.05 was considered significant.

### Study approval

Animal experiments were performed in accordance with the NIH *Guide for the Care and Use of Laboratory Animals* (National Academies Press, 2011) and approved by the UCLA Chancellor’s Animal Research Committee.

## Author contributions

SS, JAA, and MV conceived and designed research; SS, JDH, NY, MAS, and MV performed experiments; SS, JDH, NY, ZAL, and MV analyzed data; SS, JDH, JAA, JLA, ZAL, and MV interpreted results of experiments; SS, JDH, NY, and MV prepared figures; and SS, JDH, and MV drafted the manuscript. SS, NY, JDH, ZAL, MAS, JAA, JLA, and MV edited, revised, and approved the final version of the manuscript. First authorship order position was listed based on intellectual contribution to design of the study and interpretation of data.

## Supplementary Material

Supplemental data

## Version 1 01/11/2022

In-Press Preview

## Version 2 02/22/2022

Electronic publication
