# Supplementary material for: Myocardial infarction reduces cardiac nociceptive neurotransmission through the vagal ganglia
Source: JCI Insight. 2022 Feb 22;7(4):e155747. doi: 10.1172/jci.insight.155747 (PMC8876456; doi:10.1172/jci.insight.155747)
Supplement: Supplemental data [file jciinsight-7-155747-s282.pdf]

Myocardial Infarction Reduces Cardiac Nociceptive Neurotransmission through the Vagal Ganglia

Siamak Salavatian,[1,2¥] Jonathan D Hoang,[1,2¥] Naoko Yamaguchi,[1,2¥] Zulfiqar Ali Lokhandwala,[1,2] Mohammed Amer Swid,[1,2] John Andrew Armour,[1,2] Jeffrey L. Ardell,[1,2] Marmar Vaseghi[1,2*]

[1]UCLA Cardiac Arrhythmia Center and [2]UCLA Neurocardiology Research Program of Excellence, Los Angeles, CA,

¥These authors share first author position

Table 1. Hemodynamics Responses to Cardiac Interventions

		Δ HR (bpm)	Δ LVSP (mmHg)	Δ dP/dt_{max} (mmHg/s)	Δ dP/dt_{min} (mmHg/s)
**EMS**	**normal**	0.1±0.1	-3.4±1.4*	-4.0±10.7	50.6±42.8
	**MI**	-0.5±0.2	-3.0±0.9*	-8.7±9.7	58.0±19.8*
**BRADY**	**normal**	-2.0±1.5	-8.5±4.3	-99.9±57.0	667.3±240.1*
	**MI**	4.6±1.6*	-24.5±5.6*	-81.3±47.7	661.9±189.8*
**CAPS**	**normal**	-1.8±0.8	-2.4±0.8	-11.1±23.1	81.8±92.8
	**MI**	-0.1±2.6	10.2±3.1*	-49.7±27.7	-79.0±40.5
**VERAT**	**normal**	-0.4±0.1*	-0.2±2.8	18.1±22.5	-53.3±94.6
	**MI**	-1.9±1.1*	-1.2±1.8	-11.3±17.6	34.6±25.0
**VP**	**normal**	10.9±2.6*	-16.0±4.3*	-147.4±64.2*	316.5±95.9*
	**MI**	7.4±2.2*	-16.5±4.7 *	-179.4±85.4*	294.6±95.1*
**IVC**	**normal**	1.9±1.0*	-57.9±8.3*	-612.5±123.0*	1180.0±187.0*
	**MI**	6.0±2.9*	-75.2±6.9*	-946.2±102.4*	1522.0±192.8*
**AO**	**normal**	-8.3±2.6*	73.0±6.2 *	29.9±117.8	-235.9±218.0
	**MI**	-13.9±3.6*	66.6±7.7*	102.4±77.3	-54.5±131.9

Values are shown as mean ± SE for change from baseline in heart rate (HR), left ventricular end-systolic pressure (LVSP), as well as the maximum and minimum first derivatives of LV pressure (dP/dt). AO = aortic occlusion; EMS = epicardial mechanical stimulation; IVC = inferior vena cava occlusion; VP = ventricular pacing; CAPS = capsaicin; VERAT = veratridine; BRADY = bradykinin; *represent statistically significant changes in parameters from baseline.

Table 2. Antibodies used for histological analysis

					Number of Nodose Ganglia		
**Primary Antibody**	**Host**	**Dilution**	**Vendor**	**Catalog #**	**Normal**	**LAD**	**RCA**
**Anti-PIEZO2**	Rabbit Polyclonal	1:200	Neuromics	RA10109	10	10	-
**Anti-P2X3**	Rabbit Polyclonal	1:200	Thermo Fisher Scientific	PA5-72975	15	19	14
**Anti-GAD65**	Rabbit Polyclonal	1:2500	Sigma	G5038	9	10	-
**Anti-GABA**	Rabbit Polyclonal	1:1000	Immunostar	20094	11	8	-
**Anti-GABA B Receptor 1**	Mouse Monoclonal	1:300	Abcam	ab55051	20	13	-
**Anti-NOS1**	Mouse Monoclonal	1:100	Santa Cruz Biotechnology	sc-5302	16	16	13
**Anti-GFAP**	Mouse Monoclonal	1:1000	Thermo Fisher Scientific	MA5-12023	10	10	-
**Anti-CGRP**	Goat Polyclonal	1:1000	Abcam	ab36001	39	34	13

Secondary Antibody	Host	Dilution	Vendor	Catalog #
**Anti-Mouse IgG - Alexa Fluor 488**	Donkey Polyclonal	1:200	Jackson Immunoresearch	715-546-150
**Anti-Rabbit IgG - Cy3**	Donkey Polyclonal	1:200	Jackson Immunoresearch	711-166-152
**Anti-Goat IgG - Alexa Fluor 647**	Donkey Polyclonal	1:200	Jackson Immunoresearch	705-606-147
**Anti-Mouse IgG - HRP**	Donkey Polyclonal	1:200	Jackson Immunoresearch	715-036-151
**Anti-Rabbit IgG - HRP**	Donkey Polyclonal	1:200	Jackson Immunoresearch	711-036-152
**Anti-Goat IgG - HRP**	Donkey Polyclonal	1:200	Jackson Immunoresearch	705-036-147

Supplemental figure 1.

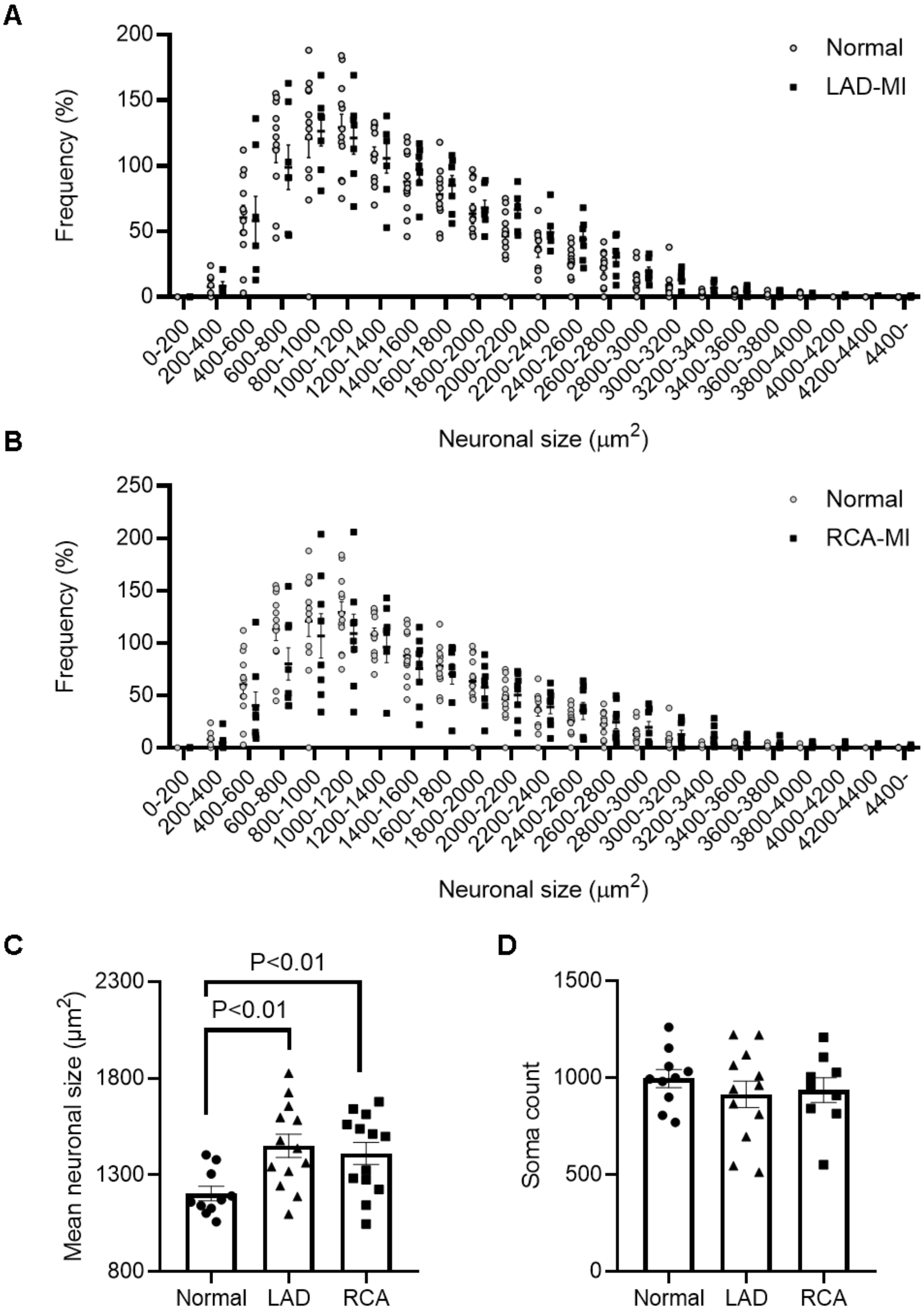

Supplemental Figure 1. *Effect of myocardial infarction and the infarct region on nodose neuronal size.* **(A)** Analysis of the distribution of neuronal sizes shows that the

number of neurons with larger sizes is increased in LAD-MI pigs (n = 7 ganglia) compared to normal pigs (n = 12 ganglia). **(B)** Neuronal size distribution in normal and RCA infarcted animals is shown. The number of neurons with larger sizes is higher in the RCA-MI pigs (n = 8 ganglia) than normal pigs (n = 12 ganglia). **(C)** Compiled data across all sizes shows that the mean nodose neuronal size is higher in infarcted animals (LAD: P = 0.004, n = 13 ganglia, vs. normal, n = 10 ganglia; RCA: P = 0.014, n = 13 ganglia, vs. normal, n = 10 ganglia). **(D)** There was no significant difference in the number of cells in normal animals (n = 10 ganglia) vs. LAD (n = 12 ganglia) or RCA (n = 9 ganglia) chronically infarcted animals. Data is shown as mean ± SE. Unpaired two-tailed Student's t-tests with the false discovery rate corrected by the Benjamini-Hochberg method was used for comparisons of ganglia from normal vs. infarcted animals.

Supplemental Figure 2.

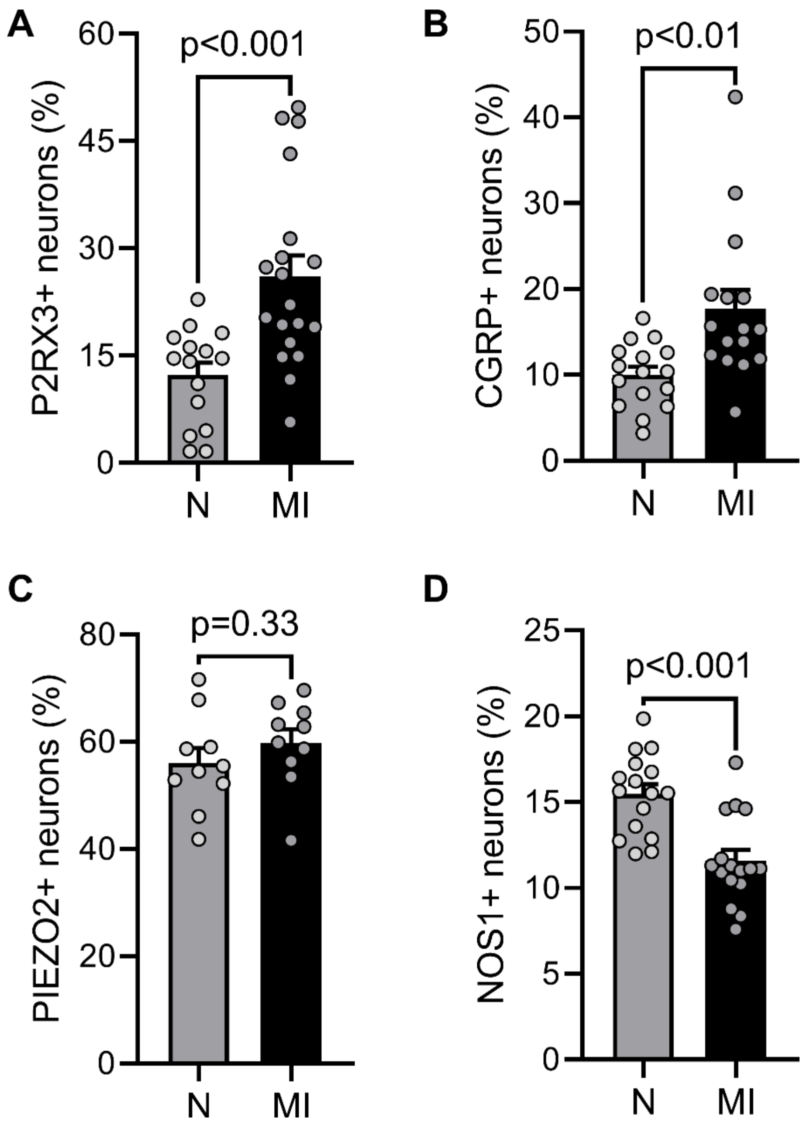

Supplemental Figure 2. ***Immunohistochemical assessment of neural remodeling after MI (analysis by nodose ganglion).*** Percentage of neurons in normal and MI nodose ganglia which express **(A)** P2RX3 ($P < 0.001$) and **(B)** CGRP ($P = 0.003$) is increased, while the expression of **(C)** PIEZO2 ($P = 0.33$) is unchanged, and **(D)** NOS1 expression is reduced ($P < 0.001$). $n = 15$ nodose ganglia from normal animals and $n = 19$ nodose ganglia from LAD-infarcted animals for P2RX3; $n = 16$ nodose ganglia from normal and MI animals for CGRP and NOS1; $n = 10$ nodose ganglia per group for PIEZO2. Data are shown as mean ± SE; unpaired, two-tailed Student's *t*-test used for comparison of MI and normal animals. N = normal animals, MI = animals with chronic LAD myocardial infarction.

Supplemental Figure 3.

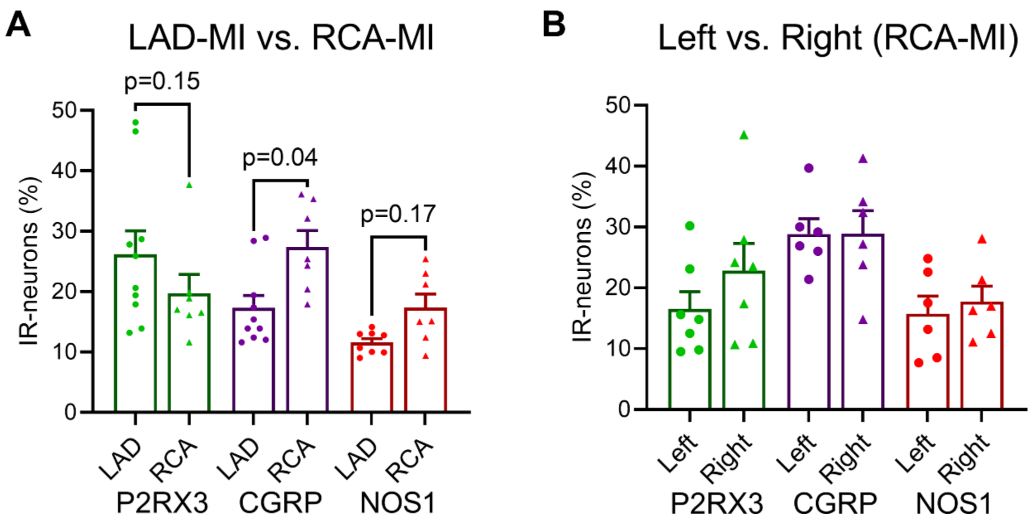

Supplemental Figure 3. ***Effect of infarcted region on remodeling of the nodose ganglia following myocardial infarction.*** **(A)** Percentage of nodose ganglia neurons from LAD and RCA infarcted animals which express P2RX3 (*N* = 10 LAD infarcted pigs, *N* = 7 RCA infarcted pigs), CGRP (*N* = 10 LAD infarcted pigs, *N* = 7 RCA infarcted pigs), and NOS1 (*N* = 8 LAD infarcted pigs and *N* = 7 RCA infarcted pigs) is shown. There was no difference in the expression profiles of nodose ganglia neurons from LAD infarcted vs. RCA infarcted animals with regards to increases in P2RX3 (*P* = 0.15) or decreases in NOS1 (*P* = 0.17) expression. RCA infarcted animals showed modestly higher expression of CGRP (*P* = 0.04) compared to LAD infarcted animals. **(B)** A comparison of right vs. left nodose ganglia neural expression profiles for P2RX3 (*P* = 0.52; *n* = 7 pairs of nodose), CGRP (*P* = 0.98; *n* = 6 pairs of nodose) and NOS1 (*P* = 0.57; *n* = 6 pairs of nodose) showed no significant differences in the expression profiles of right vs. left nodose ganglia, indicating that both the left and right-sided ganglia are affected by myocardial infarction. Data is shown as mean ± SE. (A) Unpaired or (**B**) paired, two-tailed Student's *t*-tests with the false discovery rate corrected by the Benjamini-Hochberg method were used for analysis.

Supplemental Figure 4.

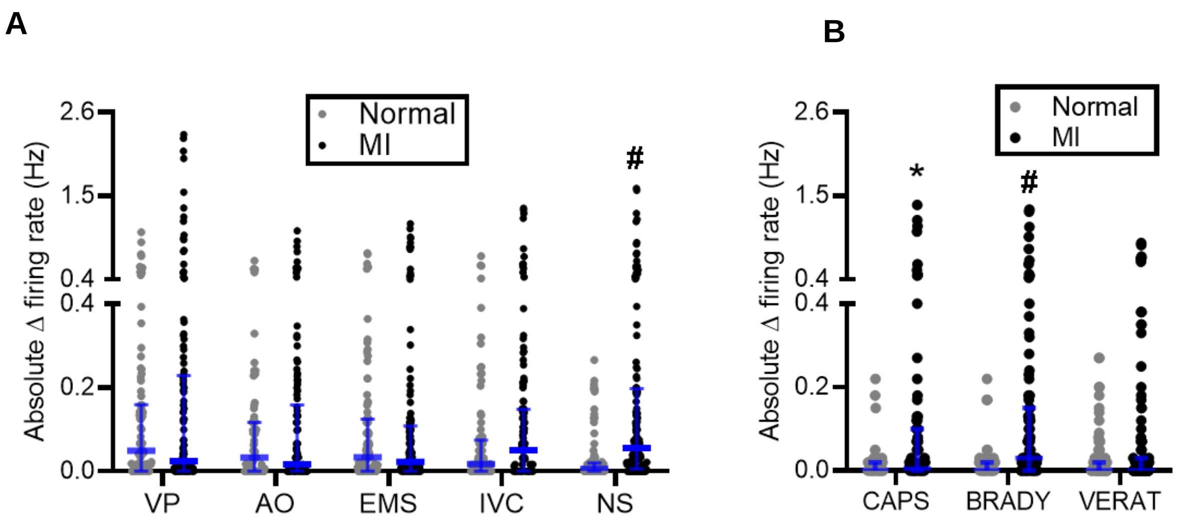

Supplemental Figure 4. ***Nodose neural responses to specific cardiac interventions.*** **(A)** Absolute changes in the firing rates of neurons (normal: 97 neurons, RCA-MI: 133 neurons) to each cardiac stressor and **(B)** to each specific chemical are also shown (number of neurons for normal animals: VP = 93, AO = 91, EMS = 94, IVC: 93, NS: 93, CAPS: 58, BRADY: 58, VERAT: 93; number of neurons for infarcted animals: VP = 129, AO = 122, EMS = 121, IVC = 128, NS = 120, CAPS = 72 BRADY = 117, VERAT = 117 neurons). Wilcoxon signed-rank test with correction for multiple comparisons was used to compare firing rates of neurons in normal vs. MI animal. *$0.01 < P \leq 0.05$, #$P \leq 0.001$ compared to normal animals.

Supplemental Figure 5.

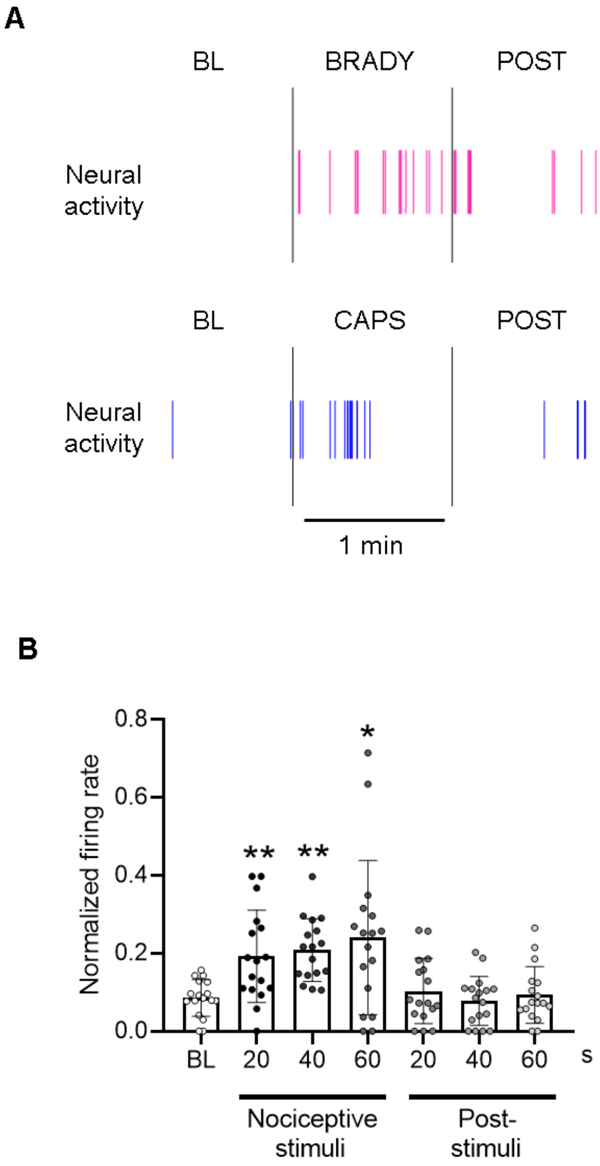

Supplemental Figure 5. ***Temporal profile of neuronal responses in normal animals demonstrates excitatory responses during application of nociceptive chemicals.*** **(A)** Representative excitatory responses to bradykinin (BRADY) and capsaicin (CAPS) of two nodose neurons from a normal animal is shown. **(B)** Quantified temporal responses in firing rates of neurons (n = 17) from normal animals showed an increase in firing rates upon epicardial application of nociceptive chemicals that subsided after removal of the stimulus. Data is shown as mean ± SE. Dunn's multiple comparison tests were used to compare the normalized firing rate vs. baseline (BL). **$P \leq 0.001$ and *$P \leq 0.05$ vs. baseline.

Supplemental Figure 6.

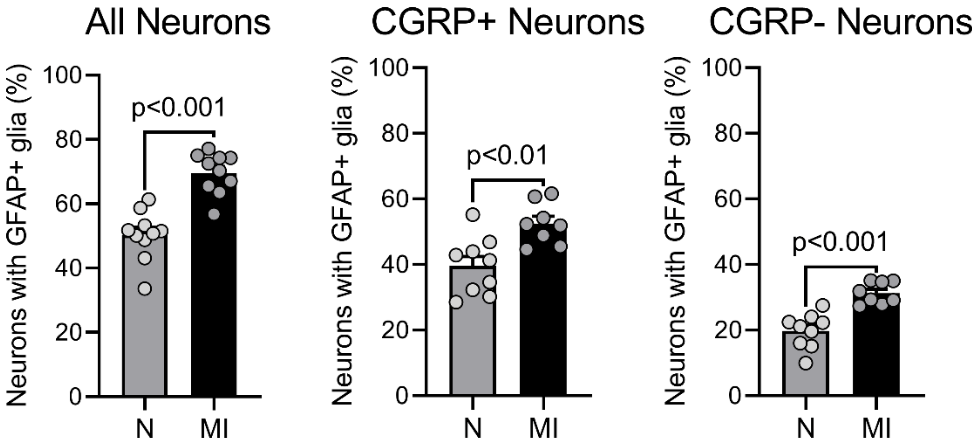

Supplemental Figure 6. ***Non-selective augmentation of nodose ganglia satellite glial cell activation following myocardial infarction involving the LAD coronary artery (analysis by nodose ganglion).*** Percentage of neurons surrounded by GFAP+ satellite glial cells in normal and MI nodose ganglia as a subset of all neurons ($P < 0.001$), CGRP+ neurons ($P = 0.004$) and CGRP- neurons ($P < 0.001$) shows that although glial activation occurs throughout the nodose ganglia of the animals after myocardial infarction, this response is not specific to CGRP expressing/nociceptive neurons. $n = 10$ nodose ganglia per group for all neurons (normal and LAD-MI), $n = 9$ nodose ganglia for CGRP+ neurons and CGRP- neurons in normal animals, $n = 8$ nodose ganglia for CGRP+ neurons and CGRP- neurons in LAD-MI animals. Data is shown as mean ± SE. Unpaired, two-tailed Student's *t*-test was used for MI vs. normal group comparisons.

Supplemental Figure 7.

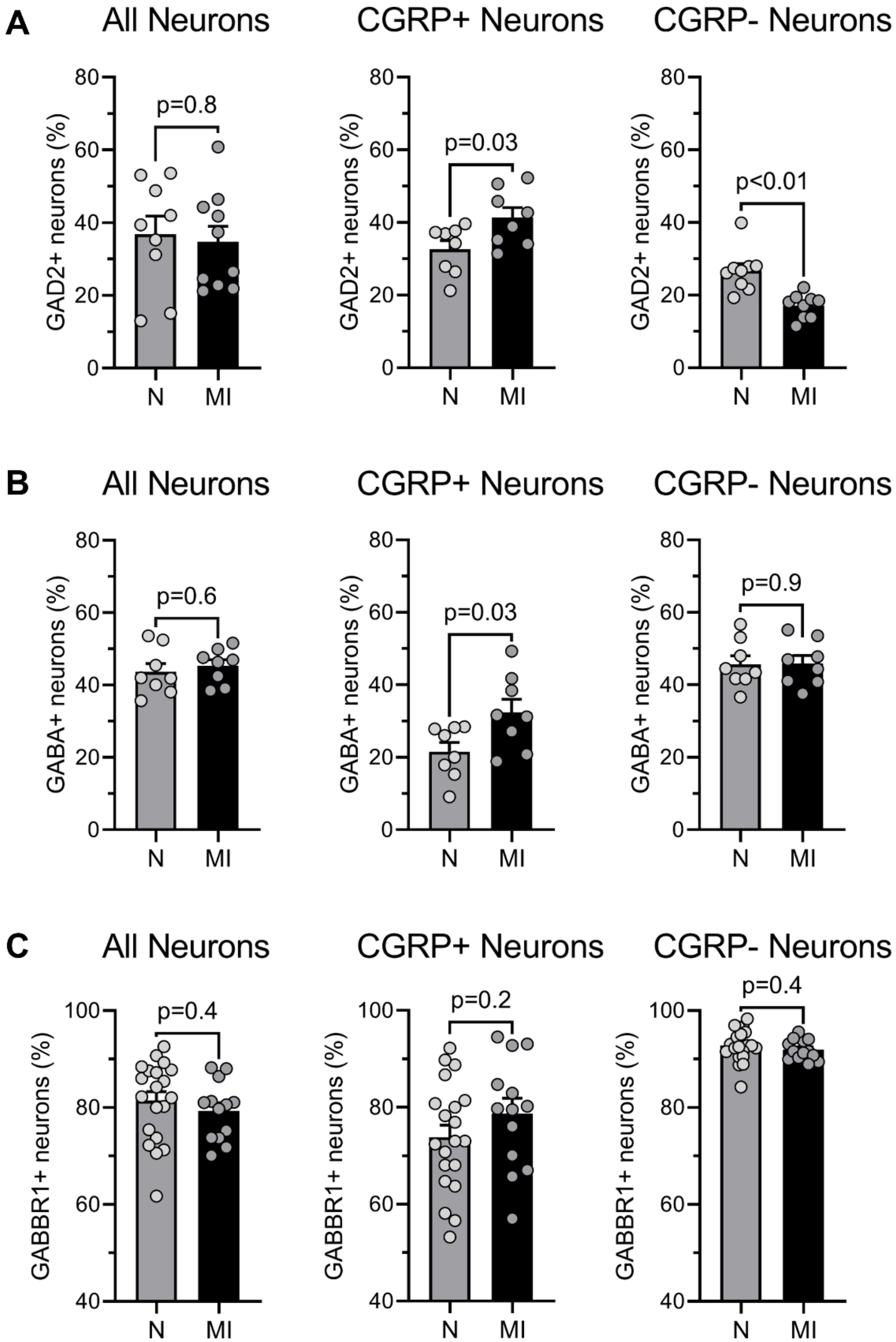

Supplemental Figure 7. ***Upregulation of inhibitory, GABAergic expression in nodose ganglia neurons co-expressing CGRP after myocardial infarction***

***involving the coronary LAD artery (analysis by nodose)**. **(A)** Percentage of GAD2+ neurons in normal and MI nodose ganglia as a subset of all neurons as well as those co-expressing CGRP and not expressing CGRP (CGRP-). The percentage of neurons that co-express CGRP and GAD2 are increased after MI. **(B)** Percentage of all neurons expressing GABA in normal and MI animals as well as the percentage co-expressing CGRP is shown, as analyzed both by animal and by ganglion. The percentage of neurons co-expressing CGRP and GABA is increased after MI. **(C)** Percentage of neurons with GABBR1+ expression in normal and MI nodose ganglia as a subset of all neurons, CGRP+ neurons, and CGRP- neurons shows no difference in the expression of GABA type B receptors after MI. *n* = 9 normal nodose and 10 MI nodose ganglia for GAD2 in all neurons. *n* = 8 normal nodose and MI nodose ganglia for GAD2+ CGRP+ neurons. *n* = 9 normal nodose and MI nodose ganglia for GAD2+ CGRP- neurons. *n* = 8 normal nodose and MI nodose ganglia for GABA. *n* = 20 normal nodose and *n* = 13 MI nodose ganglia for GABBR1. Data is shown as mean ± SE. Unpaired, two-tailed Student's *t*-test was used for comparison of normal and MI animals and ganglia.

Supplemental Figure 8.

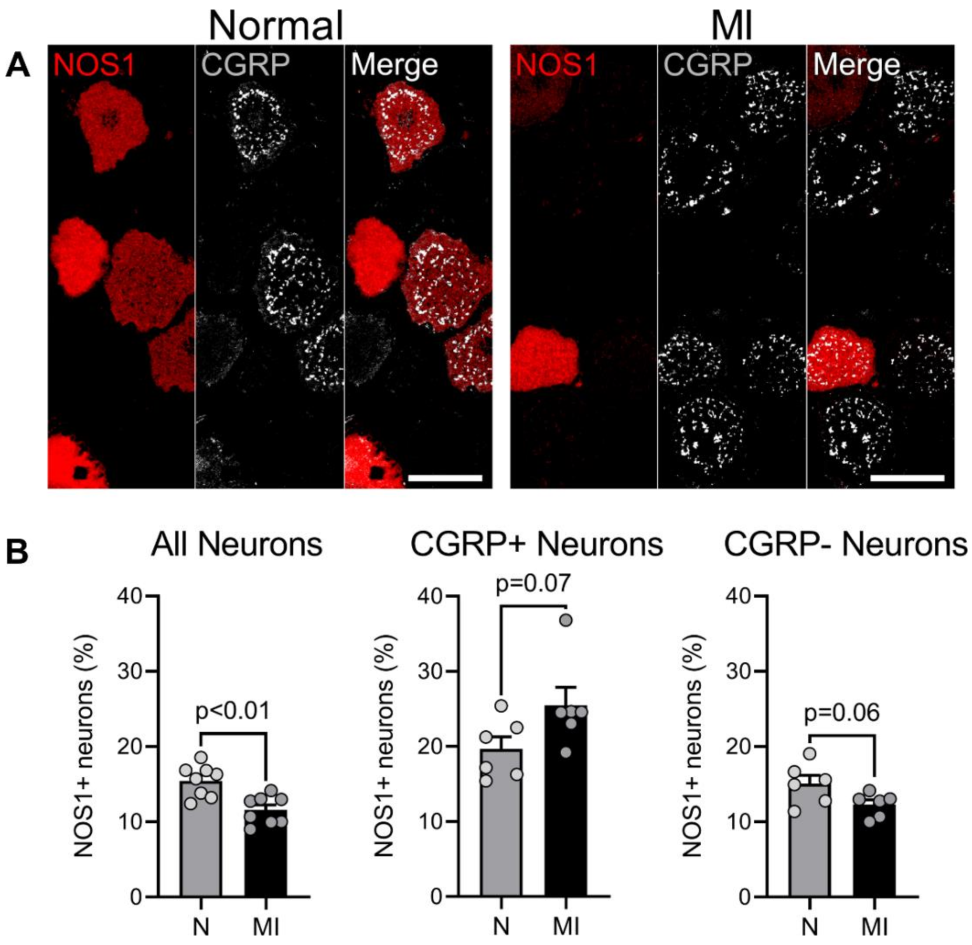

Supplemental Figure 8. ***Non-selective loss of neuronal nitric oxide synthase expression in the porcine nodose ganglia.*** **(A)** Representative images of nodose ganglia from normal (N; left) and LAD-infarcted (MI; right) animals stained for NOS1 (red) and CGRP (white) are shown in the upper panels. **(B)** Quantified expression of NOS1 in normal and MI animals (N = 8 pigs per group) and co-expression with CGRP+ neurons (P = 0.07) and CGRP- neurons (P = 0.06) (N = 6 pigs per group for co-expression) are shown. Although global expression of NOS1 is significantly reduced, the reduction is not specific to CGRP-expressing neurons. Data is shown as mean ± SE. Unpaired, two-tailed Student's *t*-test was used for comparison of normal and MI groups. Scale bars are 50 µm.
